# ScIsoX: a multidimensional framework for measuring isoform-level transcriptomic complexity in single cells

**DOI:** 10.1186/s13059-025-03758-5

**Published:** 2025-09-22

**Authors:** Siyuan Wu, Ulf Schmitz

**Affiliations:** 1 Computational Biomedicine Lab, College of Science and Engineering, James Cook University, Townsville, QLD Australia; 2 Centre for Tropical Bioinformatics and Molecular Biology, James Cook University, Cairns, QLD Australia; 3 School of Mathematics, Monash University, Melbourne, Victoria Australia; 4 Centenary Institute, The University of Sydney, Camperdown, New South Wales Australia

**Keywords:** Isoform-resolved transcriptomics, Single-cell isoform sequencing, Alternative splicing, Isoform analysis

## Abstract

Single-cell isoform analysis enables high-resolution characterization of transcript expression, yet analytical frameworks to systematically measure transcriptomic complexity are lacking. Here, we introduce ScIsoX, a computational framework that integrates a novel hierarchical data structure, a suite of complexity metrics, and dedicated visualization tools for isoform-level analysis. ScIsoX supports systematic exploration of global and cell-type-specific isoform expression patterns arising from alternative splicing, revealing multidimensional complexity signatures across diverse datasets—insights often missed by conventional gene-level approaches. We demonstrate the utility of ScIsoX across multiple real-world single-cell isoform sequencing datasets, showcasing its potential as a general framework for transcriptomic complexity analysis.

**Supplementary information:**

The online version contains supplementary material available at 10.1186/s13059-025-03758-5.

## Background

Alternative splicing dramatically expands the functional repertoire of eukaryotic cells by generating diverse transcript isoforms from a limited number of genes. Recent advances in single-cell isoform analysis have enabled comprehensive characterization of transcript diversity at unprecedented resolution. Two complementary approaches are now available: short-read methods, which offer high throughput but with limited isoform resolution, and long-read sequencing technologies, which provide full-length transcript characterization at lower throughput [1, 2]. However, analytical frameworks for measuring and interpreting the multidimensional nature of transcriptomic complexity at single-cell resolution do not exist for either platform. This represents a missed opportunity to leverage the additional layers of information provided by isoform-resolved data, which this study aims to address.

Current approaches for analyzing single-cell isoform data face three major challenges. First, conventional data structures present limitations for multidimensional complexity analysis. Gene-by-cell count matrices inherently fail to capture the complexity and variability of isoform usage across genes, while transcript-by-cell matrices with gene IDs as metadata, though more popular and widely adopted in Nanopore and PacBio software, require repeated metadata lookups and data reorganization for within-gene complexity operations to identify which transcripts belong to the same gene during analysis, causing computational inefficiency. Second, attempts to merge gene-level and isoform-level count matrices into a “cell $\times$ gene $\times$ isoform” tensor necessitate extensive zero padding to accommodate gene-specific variability in isoform numbers, resulting in sparse 3D tensors with excessive memory demands. Third, while existing analytical methods excel at isoform discovery and quantification [3, 4], they lack comprehensive metrics that address fundamental questions about the organizing principles governing isoform expression patterns across cells and cell types.

## Results and discussion

**Fig. 1:**
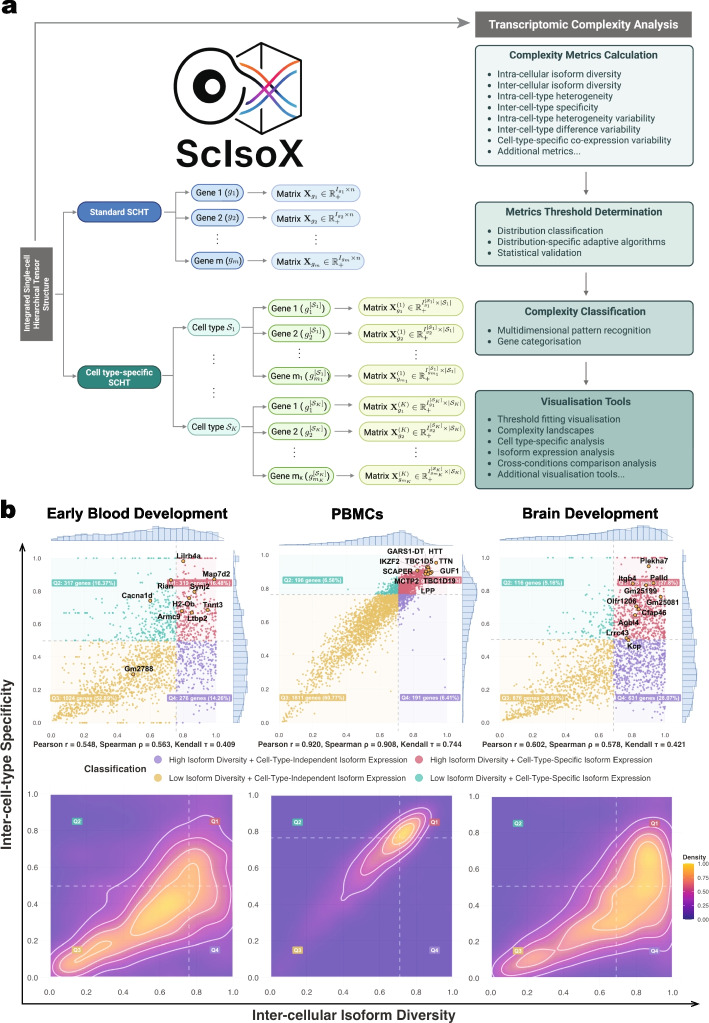
ScIsoX overview and comparison of complexity landscapes across biological systems. **a** Core of the ScIsoX computational framework showing the interconnected components of data structure and analytical processing. Left: SCHT construction organizes isoform expression data into gene-specific sub-tensors; right: ScIsoX’s analytical pipeline progressing from complexity metrics to biological insights. Created with BioRender.com. **b** Complexity landscapes in mouse early blood development, human peripheral blood mononuclear cells, and mouse brain development datasets, illustrating the relationship between selected complexity dimensions. Top: the visualized complexity space reveals gene distribution across four quadrants, with annotated genes of interest that demonstrate characteristic complexity signatures; bottom: density contour maps revealing system-specific clustering patterns, demonstrating how complexity distributions vary across different biological contexts

To address the challenges in single-cell isoform analysis, we introduce ScIsoX, a computational framework that implements (i) a novel Single-Cell Hierarchical Tensor (SCHT) data structure, (ii) a comprehensive suite of analytical metrics, and (iii) visualization tools for measuring transcriptomic complexity across multiple biological scales (Fig. 1a and Additional file 1: Fig. S1). At its core, the SCHT organizes isoform-level count data into gene-specific sub-tensors, where each gene is represented by an individual count matrix containing isoform-by-cell expression values. This partition-based design preserves the intrinsic hierarchy without resorting to extensive zero padding, yielding a representation that is both biologically meaningful and computationally efficient. When cell type information is integrated, the SCHT is extended to include cell types as an additional dimension. Each count matrix contains only the cells belonging to that particular cell type expressing the gene, creating a multi-level hierarchy that elegantly captures gene-isoform-cell relationships.

Building upon this structure, ScIsoX conceptualizes transcriptomic complexity through seven core metrics, each capturing a distinct dimension of isoform expression patterns (Fig. 1a and Additional file 2: Table S1). The primary dimensions include (I) intra-cellular isoform diversity (i.e., the tendency for a gene to co-express multiple isoforms within individual cells), (II) inter-cellular isoform diversity (i.e., the diversity of isoforms expressed by a gene across the whole cell population), (III) intra-cell-type heterogeneity (i.e., cell-to-cell variation in isoform usage), and (IV) inter-cell-type specificity (i.e., measure of cell-type-specific isoform usage). Three additional higher-order metrics measure variability in these patterns to determine, (V) whether cellular heterogeneity is concentrated in specific cell types, (VI) whether cell-type-specific differences occur between particular lineages, and (VII) whether isoform co-expression patterns vary across cell types. To complement these core metrics, we provide additional characterization metrics that capture specific aspects of isoform usage (Additional file 2: Table S2).

We have confirmed ScIsoX’s utility by analyzing three distinct single-cell isoform datasets surveying: (1) murine hematopoietic development via Nanopore sequencing [5], (2) murine and human brain development via Nanopore sequencing [6], and (3) human peripheral blood mononuclear cells (PBMCs) via PacBio’s Kinnex protocol [7]. These datasets represent fundamentally different biological systems while also employing distinct technical approaches to isoform sequencing. This selection enabled comprehensive evaluation of our framework’s performance and broad applicability. All datasets included cell type annotations for analysis. Our analysis revealed markedly different transcriptomic complexity patterns in these systems, highlighting the biological insights uniquely accessible through our approach.

**Fig. 2:**
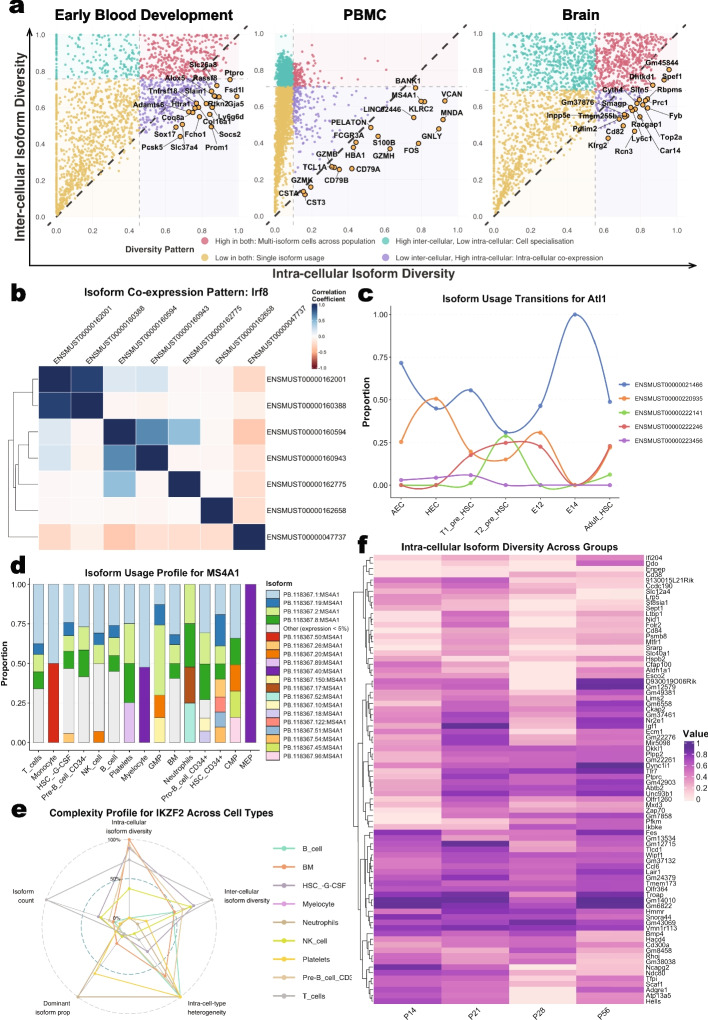
Multidimensional transcriptomic complexity analysis reveals isoform expression patterns. **a** Intra-cellular versus inter-cellular diversity analysis across three datasets. Highlighted are genes that fall below the diagonal line (i.e., where intra-cellular diversity exceeds inter-cellular diversity). **b** *Irf8* isoform co-expression correlation analysis, showing both positive and negative expression correlations between different isoforms, suggesting complex regulatory relationships. **c** *Alt1* isoform proportion transitions during mouse hematopoietic development. **d** *MS4A1* isoform usage profiles across different immune cell types in PBMCs. **e** Comparison of *IKZF2* complexity profiles across different immune cell types in PBMCs. **f** Heatmap of intra-cellular isoform diversity across brain postnatal developmental stages (days 14, 21, 28, and 56)

The transcriptomic complexity analysis implemented in ScIsoX can, for example, assess distinct isoform expression patterns (Fig. 1b). These patterns were non-randomly distributed, with murine hematopoietic development exhibiting a bimodal pattern dominated by low isoform diversity and low cell type specificity (Q3: 52.89%) with fewer genes showing cell-type-specific expression (Q1 + Q2: 32.85%) (Fig. 1b). The mouse brain development dataset exhibited a similar bimodal pattern, also demonstrating substantial diversity across quadrants with notable clusters. In contrast, the human PBMC dataset exhibited a strikingly different distribution compared to the two development datasets, showing a remarkably strong positive correlation between inter-cellular isoform diversity and inter-cell-type specificity (Fig. 1b). This tight correlation suggests that in specialized immune cells, isoform diversity is closely linked to cell-type-specific functions. Both developmental datasets showed a greater range of specificity/diversity relationships than PBMCs, reflecting greater transcriptomic heterogeneity in development compared to specialized immune cells, which require specific isoform-switching events for state transitions and to respond to cellular signals. Our framework uniquely identifies genes with interesting complexity profiles that may be overlooked by conventional single-cell data analysis. For instance, the vast majority of genes in all datasets exhibit higher inter-cellular diversity compared to intra-cellular diversity, demonstrating a fundamental principle: genes tend to express cell-type-specific isoforms rather than multiple isoforms in each cell type (Fig. 2a). However, a subset of genes with intra-cellular diversity that is higher than their inter-cellular diversity can be identified, suggesting coordinated co-expression of multiple isoforms within individual cells rather than cell-specific isoform selection. These genes may require specific interdependent isoform relationships for proper function, representing a distinct regulatory mechanism for further study. For example, while the role of *Sox17* in endothelial-to-hematopoietic transition is well-established [8, 9], the specific significance of its multiple transcript isoforms remains largely unexplored. Our analysis suggests that *Sox17* may utilize coordinated expression of multiple isoforms to achieve its diverse regulatory functions during early hematopoietic development (Additional file 1: Fig. S2).

Co-expression analysis reveals distinct patterns of coordinated isoform expression. For instance, in murine hematopoietic development, the transcription factor *Irf8*, a key interferon regulatory factor critical for myeloid lineage determination and immune cell differentiation [10], shows multiple clusters of co-expressed isoforms (Fig. 2b). A deeper analysis of the co-expression patterns in *Irf8* reveals that these patterns represent multiple, distinct modes of dynamic regulation. We identified one isoform pair (ENSMUST00000160388:*Irf8-202* vs ENSMUST00000162001:*Irf8-205*) that exhibits a significant pattern of stage-specific co-expression between a canonical protein-coding transcript and a intron-retaining variant. In contrast, another pair (ENSMUST00000047737:*Irf8-201* vs ENSMUST00000160943:*Irf8-204*) displays a mixed regulatory relationship, corresponding to a cell-type-specific switch between a full-length and a truncated protein-coding isoform. Together, these findings highlight a complex, multi-layered strategy for controlling this master transcription factor, likely involving both post-transcriptional buffering by the non-coding RNA and functional fine-tuning via protein isoform switching (see Additional file 3: Supplementary Note for the detailed case study). ScIsoX also enables tracking proportions of expressed isoforms across cell types, further highlighting dynamic changes in isoform usage, e.g., across lineages or developmental stages (Fig. 2c). In addition, ScIsoX facilitates detailed examination of genes’ cell-type-specific complexity profiles. For example, the gene *MS4A1* (encoding B-lymphocyte antigen *CD20*) exhibits distinctive isoform expression patterns across human PBMCs, with different immune cell types showing distinct isoform co-expression profiles (Fig. 2d). Notably, *MS4A1* falls below the diagonal in the diversity analysis (Fig. 2a), with multiple isoforms consistently co-expressed across most PBMC cell types (Fig. 2d), suggesting its function depends on the orchestrated interplay of specific isoform combinations across diverse immune cell types.

Unlike existing approaches that treat isoform diversity as a single dimension, ScIsoX provides both a multifaceted view of transcriptomic complexity (Fig. 2e and Additional file 1: Figs. S3 and S4) and enables researchers to generate testable hypotheses about the functional significance of alternative splicing, such as across developmental timepoints or anatomical regions. For example, ScIsoX reveals distinct patterns of intra-cellular isoform diversity across postnatal developmental stages, with clear gene clusters exhibiting stage-specific isoform expression profiles. The heatmap in Fig. 2f illustrates how certain gene groups maintain consistently high diversity (dark purple) throughout development, while others show stage-specific diversity patterns. Additionally, ScIsoX reveals distinct patterns of inter-cellular isoform diversity and inter-cell-type specificity that evolve dynamically throughout brain development and differ markedly between brain regions (Additional file 1: Figs. S5 and S6).

The structured organization of complexity metrics and hierarchical tensor format facilitates integration with complementary single-cell analysis approaches. The quantitative metrics can be correlated with differential expression patterns to identify relationships between expression levels and isoform regulation mechanisms, allowing researchers to relate changes in transcriptomic complexity with expression level alterations across conditions. The transcriptomic complexity signatures can also be correlated with DNA binding motif enrichment patterns to identify potential regulatory elements driving specific complexity profiles. Moreover, the framework’s cell type-resolved metrics can be mapped onto trajectory inference results, e.g., to characterize dynamic changes in isoform usage mechanisms during cellular differentiation processes. The classification system enables the incorporation of complexity dimensions into gene regulatory network analyses, potentially revealing how splicing regulators influence network topology and dynamics. Furthermore, these metrics support cross-species comparisons to investigate evolutionary conservation of isoform regulation patterns.

Of particular interest is the complementary relationship with differential transcript usage (DTU) methods such as DTUrtle [11] and Sierra [12]. While these established DTU approaches excel at comparative analysis, identifying statistically significant changes in transcript proportions between experimental conditions, ScIsoX addresses a fundamentally different analytical question through systematic characterization of inherent transcriptomic complexity patterns. Rather than asking “which genes show differential isoform usage between conditions?”, our framework asks “what complexity patterns characterize isoform expression within datasets?” This creates opportunities for enhanced analytical workflows where ScIsoX complexity profiles can serve as prior information to guide DTU study design, directing comparative analysis toward genes with appropriate complexity characteristics (e.g., focusing on genes with multi-isoform expression rather than binary switches), while DTU results gain deeper biological context when interpreted through ScIsoX’s complexity landscapes.

While these opportunities highlight the framework’s potential, several important factors should be considered when applying and interpreting results from ScIsoX. First and foremost, the validity of ScIsoX’s metrics is contingent upon the quality of the upstream data. A rigorous workflow before using ScIsoX is essential for reliable results. We recommend that users perform isoform quantification and filtering using established, platform-appropriate tools, and apply batch correction where the experimental design requires it. While ScIsoX includes internal filtering steps, these are intended to mitigate residual noise and do not replace the need for robust upstream quality control.

Second, the accuracy of several metrics depends on high-quality cell type annotations. While the framework is compatible with any popular single-cell clustering and annotation method, the quality of cell type definitions will affect the accuracy of specific metrics, particularly those based on cell type comparisons. In cases where cell type boundaries are ambiguous or annotations uncertain, users should exercise caution when interpreting results or focus on metrics that do not depend on cell type information.

Third, ScIsoX primarily provides descriptive metrics and exploratory visualizations for transcriptomic complexity patterns. While the co-expression analysis module include statistical tests (FDR correction, bootstrap stability), the core complexity metrics do not include *p *values for comparing across conditions. For formal statistical comparisons across conditions, we recommend exporting the complexity metrics and applying appropriate statistical tests tailored to the specific experimental design and biological questions.

Fourth, users should be aware that the analysis workflow is designed to focus on genes with detectable multi-isoform expression. Consequently, genes found to express only a single isoform after quality control are excluded from complexity analyses. This filtering step is essential for meaningful interpretation but may reduce the final number of genes under consideration. Improved sequencing quality and depth can significantly mitigate this issue by enabling more comprehensive isoform detection. If users wish to maximize the number of genes in subsequent analyses, they can increase the $n_{\text {hvg}}$ parameter during SCHT creation, though this value cannot exceed the total number of genes present in the dataset.

Finally, while the hierarchical data structure offers computational advantages for typical single-cell datasets, extremely large datasets may still require additional optimization strategies. The framework includes options for batch-wise processing and memory-efficient data handling to address these scenarios.

## Conclusions

In summary, ScIsoX establishes the first comprehensive framework for systematic measurement and visualization of isoform-level transcriptomic complexity in single-cell sequencing data across platforms. Through its novel hierarchical data structure, ScIsoX captures distinct dimensions of complexity at the gene, cell type, and cell population levels, generating isoform-level insights into transcriptome regulation often missed by conventional gene-level analyses. ScIsoX’s multidimensional complexity metrics and intuitive visualizations provide a foundation for investigating the functional roles of alternative splicing, e.g., in cell differentiation, development, and disease contexts across diverse biological systems. By using standard R objects for its core data structures and metrics, ScIsoX creates opportunities for future integration with other omics layers and analytical methods, positioning the framework as a valuable addition to the single-cell analysis ecosystem. The framework processes isoform count matrices from diverse sequencing platforms, making multidimensional complexity analysis broadly accessible, though users should consider platform-specific limitations when interpreting complexity metrics and other key factors.

## Methods

### Single-cell hierarchical tensor creation

ScIsoX introduces a novel hierarchical data structure that efficiently represents the three-dimensional relationship between genes, isoforms, and cells. Unlike conventional approaches that use either separate gene/transcript matrices, our approach organizes isoform-level data into gene-specific sub-tensors. Each gene is represented by an individual matrix containing isoform-by-cell expression values, preserving the intrinsic hierarchy without extensive zero padding. While we refer to our data structure as a “hierarchical tensor,” we intentionally diverge from the strict mathematical definition, instead adopting a biologically oriented representation specifically tailored to single-cell isoform data. This data structure emphasizes functional utility and simplicity while facilitating scalable analysis at the isoform level to directly confront the intricacy of transcriptomic complexity.

#### Quality control and normalization

Quality control and normalization are performed in ScIsoX. It requires the following inputs: (i) a gene count matrix, (ii) an isoform count matrix, (iii) a transcript annotation file, and (iv) cell metadata (optional). The framework supports both raw count matrices and pre-normalized count matrices through the input_type parameter, which can be set to either “raw_counts” or “normalised.”

For the example datasets, genes were filtered that were detected in fewer than $p_{\min }$ proportion of cells (default: 0.02) and with mean expression counts below $\varepsilon$ (default: $1 \times 10^{-4}$). All transcripts belonging to retained genes were kept to preserve complete isoform diversity information. At the cell level, we employed a data-adaptive approach to identify and exclude low-quality cells and potential doublets based on the distribution of detected genes using the plot_genes_per_cell_distribution() and recommend_qc_parameters() functions. Cells with fewer than $n_{\min }$ genes (default: 200) or more than $n_{\max }$ genes (default: 20,000) were excluded.

For normalization, when input_type = ”raw_counts”, retained count data were normalized using counts per million (CPM) with subsequent logarithmic transformation:

1 $$\begin{aligned} x_{gi} = \log _2\left( \frac{c_{gi}}{\sum _{j=1}^{G}c_{gj}} \times 10^6 + 1\right) , \end{aligned}$$

where $c_{gi}$ represents the raw count for feature *g* in cell *i*, and *G* being the total number of features (genes or transcripts). When input_type = ”normalised”, the input count matrices are assumed to be already pre-normalized (e.g., TPM or FPKM), and only logarithmic transformation is applied:

2 $$\begin{aligned} x_{gi} = \log _2(c_{gi} + 1), \end{aligned}$$

where $c_{gi}$ represents the raw count for feature *g* in cell *i*.

#### Identification of highly variable genes

To prioritize computational resources on genes exhibiting biologically meaningful variation, we implemented a dispersion-based selection of highly variable genes (HVGs). For each gene *g*, ScIsoX calculates the variance-to-mean ratio based on their normalized expression counts. Genes are ranked by their dispersion values, and the top $n_{\text {HVG}}$ genes (default: 3000) are selected for subsequent analysis. This approach effectively identifies genes with significant biological variability while excluding stably expressed housekeeping genes and technical noise. If a greater number of genes is desired for inclusion, $n_{\text {HVG}}$ can be increased up to the total number of genes in the dataset.

#### Mathematical formulation of the SCHT data structure

We developed a hierarchical structure to represent single-cell isoform data. Let $\mathcal {C} = \{c_1, c_2, \ldots , c_n\}$ be the set of all cells after quality control and filtering, and $\mathcal {G} = \{g_1, g_2, \ldots , g_m\}$ be the set of HVGs. For each HVG $g \in \mathcal {G}$ with $I_g$ isoforms measured across *n* cells, we define a gene-specific expression matrix:

3 $$\begin{aligned} \textbf{X}_g \in \mathbb {R}_+^{I_g \times n}, \end{aligned}$$

where each element $x_{ij} \in \textbf{X}_g$ represents the normalized expression of isoform *i* of gene *g* in cell *j* (see Fig. 1a for visual representation of this hierarchical structure). Note that $I_g$ represents the total number of isoforms for gene *g*, and different genes may have different numbers of isoforms. The standard SCHT data structure is defined as the collection $\mathcal {T} = \{(g, \textbf{X}_g) \mid g \in \mathcal {G}\}$. Cell-type-specific sub-tensors are created when cell type information is available. We partition the filtered cell set $\mathcal {C}$ into *K* non-overlapping cell types $\mathcal {S} = \{\mathcal {S}_1, \mathcal {S}_2, \ldots , \mathcal {S}_K\}$. Note that not all genes are expressed in every cell type. For each HVG $g^{[S_k]} \in \mathcal {G}$ that is expressed in cell type $\mathcal {S}_k \in \mathcal {S}$, where $k = 1, 2, \cdots , K$, we denote $I_g^{[\mathcal {S}_k]}$ as the number of isoforms of gene *g* that are expressed in cell type $\mathcal {S}_k$. We then define a cell-type-specific expression matrix $\textbf{X}_g^{(k)} \in \mathbb {R}_+^{I_g^{[\mathcal {S}_k]} \times |\mathcal {S}_k|}$ containing columns corresponding to cells of type $\mathcal {S}_k$. The integrated SCHT data structure with cell-type-specific structure is then defined as follows,

4 $$\begin{aligned} \mathcal {T}_{\text {integrated}} = \{(g, \textbf{X}_g, \{\textbf{X}_g^{(1)}, \textbf{X}_g^{(2)}, \ldots , \textbf{X}_g^{(K)}\}) \mid g \in \mathcal {G}\}. \end{aligned}$$

This hierarchical representation facilitates comprehensive analysis of transcriptomic complexity.

### Multi-dimensional transcriptomic complexity framework

We developed a comprehensive transcriptomic complexity analysis framework that quantifies key aspects of transcriptomic complexity, focusing on a core set of metrics that capture the essential dimensions of isoform expression patterns (Additional file 2: Table S1).

#### I: intra-cellular isoform diversity

To quantify isoform diversity within individual cells, ScIsoX computes a weighted Shannon entropy measure for each gene. For cell $c_j \in \mathcal {C}$ expressing gene $g \in \mathcal {G}$, the normalized Shannon entropy is defined as

5 $$\begin{aligned} H_j = \frac{-\sum _{i=1}^{I_g} p_{ij}\log _2(p_{ij})}{\log _2(n_j)}, \end{aligned}$$

where $p_{ij} = x_{ij}/\sum _{k=1}^{I_g}x_{kj}$ represents the proportion of gene expression attributed to isoform *i* in cell $c_j$, and $n_j$ is the number of isoforms detected in that cell. To account for expression magnitude, we compute a weighted mean across cells as intra-celluar isoform diversity,

6 $$\begin{aligned} \text {IDI}_{\text {intra}}(g) = \frac{\sum _{j=1}^{n} w_j H_j}{\sum _{j=1}^{n} w_j}, \end{aligned}$$

where $w_j = \sum _{i=1}^{I_g}x_{ij}$ is the total expression of gene *g* in cell $c_j$. This metric measures the tendency for genes to co-express multiple isoforms within individual cells.

#### II: inter-cellular isoform diversity

To assess isoform diversity at the cell population level, ScIsoX computes the Shannon entropy of the mean isoform expression proportions across all cells, normalized by the maximum possible entropy as

7 $$\begin{aligned} \text {IDI}_{\text {inter}}(g) = \frac{-\sum _{i=1}^{I_g} \bar{p}_i\log _2(\bar{p}_i)}{\log _2(I_g)}, \end{aligned}$$

where $\bar{p}_i = \bar{x}_i/\sum _{k=1}^{I_g}\bar{x}_k$, and $\bar{x}_i = \frac{1}{n}\sum _{j=1}^{n}x_{ij}$ is the mean expression of isoform *i* across all cells. This metric quantifies the overall diversity of isoforms used across the entire cell population.

#### III: intra-cell-type heterogeneity

To quantify cell-to-cell variation in isoform usage within a given cell type, ScIsoX computes the average Jensen-Shannon distance between cells. For a gene $g \in \mathcal {G}$ expressed in cell type $\mathcal {S}_k \in \mathcal {S}$ with $n_k$ cells, intra-cell-type heterogeneity is defined as

8 $$\begin{aligned} \text {Het}_k(g) = \frac{2}{n_k(n_k-1)} \sum \limits _{i=1}^{n_k-1}\sum \limits _{j=i+1}^{n_k}\sqrt{\frac{1}{2}D_{KL}(p_i||m_{ij}) + \frac{1}{2}D_{KL}(p_j||m_{ij})}, \end{aligned}$$

where $p_i$ and $p_j$ are the isoform proportion vectors for cells $c_i \in \mathcal {C}$ and $c_j \in \mathcal {C}$ in the cell type $\mathcal {S}_k$, $m_{ij}$ is their average distribution, and $D_{KL}$ is the Kullback-Leibler divergence. The overall intra-cell-type heterogeneity is calculated as the mean across all cell types where the gene is expressed. This metric measures cell-to-cell variation in isoform usage within each cell type. It reveals whether cells of the same type use isoforms consistently.

#### IV: inter-cell-type specificity

To assess how distinctly a gene deploys its isoforms across different cell types, ScIsoX computes the average Jensen-Shannon distance between cell-type-specific isoform profiles. For a gene $g \in \mathcal {G}$ expressed in *S* cell types, inter-cell-type specificity is defined as

9 $$\begin{aligned} \text {Spec}(g) = \frac{2}{S(S-1)} \sum \limits _{i=1}^{S-1}\sum \limits _{j=i+1}^{S}\sqrt{\frac{1}{2}D_{KL}(\bar{p}_i||m_{ij}) + \frac{1}{2}D_{KL}(\bar{p}_j||m_{ij})}, \end{aligned}$$

where $\bar{p}_i$ and $\bar{p}_j$ are the vectors of mean isoform proportions for cell types $\mathcal {S}_i \in \mathcal {S}$ and $\mathcal {S}_j \in \mathcal {S}$. Higher values indicate cell-type-specific isoform usage patterns, suggesting specialized functional roles across different cell populations.

#### V: intra-cell-type heterogeneity variability

To determine whether cellular heterogeneity is concentrated in specific cell types, ScIsoX computes the coefficient of variation (CV) of intra-cell-type heterogeneity values across cell types. For a gene $g \in \mathcal {G}$ expressed in *S* cell types, this variability is defined as

10 $$\begin{aligned} \text {HetVar}(g) = \frac{\sigma (\{\text {Het}_1(g), \text {Het}_2(g), \ldots , \text {Het}_S(g)\})}{\mu (\{\text {Het}_1(g), \text {Het}_2(g), \ldots , \text {Het}_S(g)\})}, \end{aligned}$$

where $\sigma$ and $\mu$ represent the standard deviation and mean, respectively. High values indicate that some cell types have higher internal heterogeneity than others, suggesting targeted subpopulation structure or regulatory plasticity within specific lineages.

#### VI: inter-cell-type difference variability

To assess whether isoform usage differences are concentrated between specific cell type pairs, ScIsoX computes the CV of pairwise Jensen-Shannon distances. For a gene $g \in \mathcal {G}$ expressed in *S* cell types, this variability is defined as

11 $$\begin{aligned} \text {DiffVar}(g) = \frac{\sigma (\{\text {JS}_{1,2}, \text {JS}_{1,3}, \ldots , \text {JS}_{S-1,S}\})}{\mu (\{\text {JS}_{1,2}, \text {JS}_{1,3}, \ldots , \text {JS}_{S-1,S}\})}, \end{aligned}$$

where $\text {JS}_{i,j}$ is the Jensen-Shannon distance between cell types *i* and *j*. High values indicate that certain cell type pairs exhibit particularly divergent isoform usage patterns, suggesting lineage-specific splicing regulation or functional specialization between specific cell populations.

#### VII: cell-type-specific co-expression variability

To evaluate whether a gene is subject to different co-expression patterns across cell types, ScIsoX computes the CV of mean intra-cellular diversity across cell types. For a gene $g \in \mathcal {G}$ expressed in *S* cell types, this variability is defined as

12 $$\begin{aligned} \text {CoExpVar}(g) = \frac{\sigma (\{\text {IDI}_{\text {intra}_1}(g), \text {IDI}_{\text {intra}_2}(g), \ldots , \text {IDI}_{\text {intra}_S}(g)\})}{\mu (\{\text {IDI}_{\text {intra}_1}(g), \text {IDI}_{\text {intra}_2}(g), \ldots , \text {IDI}_{\text {intra}_S}(g)\})}, \end{aligned}$$

where $\text {IDI}_{\text {intra}_k}(g)$ is the mean intra-cellular isoform diversity of gene *g* in cell type $\mathcal {S}_k$ (i.e., the tendency for genes to co-express multiple isoforms across cell type $\mathcal {S}_k$). High values indicate that a gene exhibits dramatically different co-expression patterns in different cellular contexts, suggesting context-dependent regulation of isoform co-expression.

#### Additional complexity metrics

ScIsoX computes a range of supplementary metrics to further characterize isoform expression pattern. These additional metrics are detailed in Additional file 2: Table S2.

### Optimal threshold determination for complexity classification

The ScIsoX framework implements an advanced multi-stage statistical pipeline to determine optimal classification thresholds for each complexity dimension. This methodology addresses the challenges of analyzing heterogeneous distribution patterns observed in transcriptomic complexity metrics.

#### Distribution-aware preprocessing

For each complexity metric, we first apply distribution-aware preprocessing to identify the underlying distribution characteristics. This preprocessing phase employs multiple statistical approaches:

1. **Distribution classification:** Each metric’s distribution is classified into one of several categories: multimodal, zero-inflated, extremely skewed, moderately skewed, or unimodal using a comprehensive multi-method approach. For multimodality detection, we employ three complementary methods, namely Hartigan’s dip test for statistical significance, kernel density estimation with adaptive bandwidth selection for peak/valley analysis, and Gaussian mixture modeling with Bayesian Information Criterion for component separation. Skewness is assessed using moment-based calculations with distinct thresholds for moderate and extreme cases.
2. **Zero-inflation detection:** An adaptive histogram-based approach is used to identify zero-inflated distributions. This method calculates a data-dependent near-zero threshold (based on range and interquartile range), determines optimal bin width using the Freedman-Diaconis rule, and analyzes the ratio between first and second bins to detect significant zero-inflation. For identified zero-inflated distributions, we further characterize the non-zero component, testing for multimodality and skewness to determine appropriate transformation strategies.
3. **Transformation application:** When necessary, Yeo-Johnson transformations are applied with optimized parameters to normalize extremely skewed distributions while preserving their essential characteristics for threshold determination.

#### Distribution-specific threshold algorithms

Based on the identified distribution type, specialized algorithms are employed to determine optimal thresholds, with a hierarchical fallback strategy to ensure robust results (Additional file 1: Figs. S7 and S8):

1. **For multimodal distributions:** our algorithm first attempts to identify inflection points in the density curve, followed by mixture model-based component separation if needed. For distributions with clear valleys between modes, it calculates the optimal separation threshold based on relative depths and positions of these valleys.
2. **For extremely skewed distributions:** our algorithm avoids extreme tails by focusing on the central mass of the distribution, using inflection point and curvature analysis to identify natural separation points.
3. **For zero-inflated distributions:** the non-zero component is extracted and analyzed separately, using either gap detection (for significant discontinuities), mixture modeling (for multimodal non-zero components), or adaptive percentiles based on the skewness of the non-zero component.
4. **For moderately skewed and unimodal distributions:** our algorithm employs a combination of density curve analysis, distribution moments, and weighted mixtures of normal distributions to identify optimal decision boundaries.

Each method includes reliability assessment, with automatic fallback to simpler techniques when necessary. This adaptive approach ensures robust threshold determination across diverse distribution patterns encountered in complexity metrics.

#### Statistical validation framework

The reliability of determined thresholds is assessed through a comprehensive validation framework:

1. **Bootstrap stability assessment:** ScIsoX performs 100 (adjustable) bootstrap iterations, recalculating the threshold for each resampled dataset. This provides confidence intervals, standard deviations, and coefficients of variation that inform reliability scores, with higher weight given to stable thresholds.
2. **K-fold cross-validation:** For datasets with sufficient samples, ScIsoX performs stratified k-fold cross-validation to assess threshold consistency across different subsets of the data. The cross-validation coefficient of variation is integrated into the final reliability assessment.
3. **Distribution-specific reliability adjustment:** Initial reliability scores derived from the primary threshold method are adjusted based on distribution characteristics, with higher penalties for problematic distributions and distributions with limited supporting data.
4. **Sanity checking:** Final thresholds undergo verification against the data distribution’s quantiles to ensure they are reasonable, with automated adjustments applied when necessary to prevent threshold placement in extreme distribution tails.

### Classification system

Based on the seven core metrics, we developed a multi-dimensional classification system that categorizes genes according to their complexity profiles (Additional file 2: Table S3). For each dimension, genes are classified into biologically meaningful categories based on the thresholds derived from our distribution-specific threshold algorithms:

1. Intra-cellular isoform diversity is classified as “Strong Isoform Co-expression” or “Weak Isoform Co-expression”
2. Inter-cellular isoform diversity is classified as “High Isoform Diversity” or “Low Isoform Diversity” (see, for example, in Fig. 1b)
3. Intra-cell-type heterogeneity is classified as “High Cellular Heterogeneity” or “Low Cellular Heterogeneity”
4. Inter-cell-type specificity is classified as “Cell-Type-Specific Isoform Expression” or “Cell-Type-Independent Isoform Expression” (see, for example, in Fig. 1b)
5. Intra-cell-type heterogeneity variability is classified as “Variable Heterogeneity Across Cell Types” or “Consistent Heterogeneity Across Cell Types”
6. Inter-cell-type difference variability is classified as “High Cell-Type Distinctions” or “Low Cell-Type Distinctions”
7. Cell-type-specific co-expression variability is classified as “Cell-Type-Adaptive Co-expression” or “Cell-Type-Consistent Co-expression”

The integrated classification system enables systematic comparison of transcriptomic complexity patterns across genes and facilitates the identification of genes with interesting or unusual complexity profiles (see example in Additional file 2: Table S4). Additionally, NA values are preserved throughout this process, as they are generated when biologically meaningful conditions (such as single-isoform genes or single-cell-type expression) render certain metrics mathematically undefined (see Additional file 2: Table S5).

### Visualization and analysis features

The ScIsoX framework implements a comprehensive suite of visualization and analysis tools designed to explore and interpret multidimensional transcriptomic complexity patterns. The framework’s data structure facilitates efficient analytical workflows that enable researchers to gain biological insights from complex isoform expression patterns.

#### Core data structures and organization

The framework organizes isoform complexity data into two complementary object structures that support diverse analytical approaches. The IntegratedSCHT object encapsulates gene-level isoform expression matrices in a hierarchical structure, with both global and cell-type-specific expression patterns stored efficiently in a list-based format. The transcriptomic_complexity object contains a data frame of complexity metrics (metrics), cell-type-specific measurements (cell_type_metrics), classification thresholds, and statistical metadata. These data structures, combined with the S3 method system for object manipulation in R, enable sophisticated data exploration.

#### Analytical tools

1. **SCHT Structure Creation** via create_scht() transforms single-cell isoform expression matrices into SCHT structures, with supporting functions create_transcript_info() for GTF processing, and generate_gene_counts() for gene-level aggregation if only transcript count matrices are available. The SCHT structure efficiently organizes expression data by gene while preserving cell-specific isoform information.
2. **Complexity Metrics Calculation** through calculate_isoform_complexity_metrics() computes the seven core metrics: (i) intra-cellular isoform diversity, (ii) inter-cellular isoform diversity, (iii) intra-cell-type heterogeneity, (iv) inter-cell-type specificity, (v) intra-cell-type heterogeneity variability, (vi) inter-cell-type difference variability, and (vii) cell-type co-expression variability.
3. **Cell-Type-Specific Complexity Analysis** is automatically performed when calculate_isoform_complexity_metrics() is applied to an IntegratedSCHT object, calculating and comparing complexity metrics independently for each cell type, enabling the identification of cell types with distinctive isoform regulation patterns.
4. **Complexity Pattern Filtering** identifies genes matching specific combinations of complexity classifications across multiple dimensions using the find_complexity_pattern() function. This enables targeted discovery of genes with precise complexity signatures of interest.
5. **Gene Selection Tool** extracts genes with specific complexity characteristics using the select_genes_of_interest() function with customizable filtering criteria.
6. **Complexity Metric Comparison** extracts and compares transcriptomic complexity metrics across multiple genes using the compare_gene_metrics() function for custom visualizations or statistical analyses.
7. **Co-expression Analysis Suite** provides comprehensive isoform co-expression analysis through multiple integrated functions. Core analytical functions include (i) calculate_isoform_coexpression() for computing correlation matrices between isoforms for the whole dataset; (ii) calculate_gene_coexpression_all_celltypes() for systematic analysis of co-expression patterns across different cell types; (iii) analyse_coexpression_conservation() for identifying conserved versus cell-type-specific co-expression patterns. The related statistical validation including bootstrap stability testing (100 iterations) and false discovery rate correction is available through the interactive Shiny application; (iv) detect_isoform_switching() for identifying antagonistic isoform relationships; and (v) calculate_coexpression_stats() for comprehensive statistical summaries. The suite is able to handle mixed conservation patterns where isoform pairs show opposing correlations across cell types, preventing misinterpretation of overall statistics (Additional file 1: Fig. S9).
8. **Comprehensive Quality Control Reporting** creates comprehensive HTML or Markdown reports documenting the complete analysis workflow using the generate_qc_report() function. This report automatically summarizes key statistics from each stage of the create_scht() pipeline, including (i) initial data characteristics (e.g., number of cells, genes, and cell type distribution); (ii) the effects of QC filtering, detailing the number of features removed at each step; (iii) a summary of highly variable gene selection, including the number of genes removed due to single isoform expression; and (iv) a detailed computational performance and memory efficiency analysis, comparing the SCHT structure to other data representations (as shown in Additional files 4–6). Reports can be customized with dataset-specific naming through the dataset_name parameter. This automated reporting provides users with critical insights to build confidence in their data quality and analysis results.

#### Visualization capabilities

1. **Quality Control Visualizations** via plot_genes_per_cell_distribution() display the distribution of genes per cell with automatic threshold recommendations using the recommend_qc_parameters() function, helping users make informed decisions about quality control parameters.
2. **Distribution Threshold Fitting Plots** via plot_threshold_visualisations() visualize the distributions of complexity metrics across multiple cell types with optimal threshold determined by the algorithm (Additional file 1: Figs. S7 and S8).
3. **Complexity Landscape Visualizations** via plot_tc_landscape() generate bivariate scatter plots that position genes across two complexity dimensions with integrated marginal distributions. These plots incorporate quadrant statistics and threshold lines to identify genes with exceptional complexity profiles (Fig. 1b top). Interactive highlighting capabilities facilitate the identification of notable genes.
4. **Density Contour Maps** via plot_tc_density()overlay kernel density estimation contours on complexity landscapes to reveal clustering patterns and high-density regions in the complexity space (Fig. 1b bottom). Density contour maps employ adaptive bandwidth algorithms that accommodate varying data densities and highlight regions of biological significance through smooth visualization of gene concentration hotspots.
5. **Ridge Plots** via plot_complexity_ridges() visualize the distribution of complexity metrics through overlapping density curves (Additional file 1: Fig. S3). The implementation supports both global complexity comparisons across metrics and cell-type-specific analyses, offering a compact way to compare multiple distributions simultaneously.
6. **Complexity Radar Charts** via plot_complexity_radar() visualize the complete seven-dimensional complexity signature of individual genes or comparative profiles across multiple genes (Fig. 2e and Additional file 1: Fig. S4a). The implementation supports various normalization methods and custom axis configurations for effective comparison of complexity profiles.
7. **Multi-gene Cell-Type-specific Radar Charts** via plot_single_gene_radar_cell_type() and plot_compare_multiple_genes_radar_cell_type() facilitate the comparison of complexity profiles across multiple genes and cell types in a structured grid layout, enabling the identification of cell-type-specific regulatory patterns (Additional file 1: Fig. S4b). The implementation includes options for global or per-cell type scaling.
8. **Dual Diversity Plots** via plot_diversity_comparison() are scatter plots for intra-cellular and inter-cellular diversity metrics with diagonal reference lines indicating the theoretical equality boundary (Fig. 2a). This visualization specifically highlights genes exhibiting unusual diversity patterns, which may indicate specialized regulatory mechanisms.
9. **Co-expression Visualizations** encompass multiple complementary approaches for exploring isoform relationships. (i) **Correlation heatmaps** (Fig. 2b) are generated via plot_isoform_coexpression() using the ComplexHeatmap package [13], featuring hierarchical clustering to automatically detect modules of coordinated or mutually exclusive isoform usage. Users can select among multiple correlation methods (Pearson, Spearman, and Kendall) to accommodate different data distributions, with options to display correlation values directly. (ii) **Cell-type-specific dynamics** are visualized through plot_coexpression_across_celltypes(), which creates line plots revealing correlation changes across different cell populations. (iii) **Conservation summaries** via plot_conservation_summary() use bar charts to display the distribution of conserved, cell-type-specific, and mixed patterns. (iv) An **interactive Shiny application** via launch_coexpression_app() (Additional file 1: Fig. S9) provides real-time exploration with parameter adjustment, statistical testing results, and downloadable reports, with all heatmaps also generated using ComplexHeatmap package. Together, these visualizations facilitate discovery of complex regulatory patterns and hypothesis generation.
10. **Isoform Usage Profile Plots** via plot_isoform_profile() are stacked bar charts that display proportions of expressed isoform usage across cell types, developmental stages, or experimental conditions (Fig. 2c). These plots include automatic minor isoform grouping and customizable cell type ordering, facilitating the identification of cell-type-specific isoform preferences.
11. **Isoform Transition Plots** via plot_isoform_transitions() visualize dynamic changes in isoform usage across ordered cell types, time points, or developmental stages (Fig. 2d). This approach is particularly effective for revealing isoform switching events during differentiation processes or disease progression.
12. **Complexity Metric Heatmaps** via plot_compare_tc_complexity_heatmap() provide a comprehensive view of multiple complexity metrics across different groups or conditions (Fig. 2f). These heatmaps can be configured to show absolute values or changes between consecutive conditions, with gene selection based on variance, magnitude of change, or custom gene lists. These heatmaps are generated using the ComplexHeatmap package [13].
13. **Group Comparison Density Difference Maps** via plot_compare_tc_density_difference() calculate and visualize the density differences of genes in 2D metric space between different experimental groups or conditions (Additional file 1: Figs. S5 and S6). These visualizations help identify regions where gene distributions shift across conditions, revealing patterns of coordinated complexity changes in response to experimental manipulations.

All analysis and visualization functions support comprehensive parameterization while maintaining computational efficiency for large-scale datasets.

### Computational performance

To provide quantitative evidence of our approach’s efficiency, we performed comprehensive sparsity analyses across three diverse datasets (see Additional file 2: Table S6). A naive 3D tensor implementation would require extensive zero-padding to accommodate the maximum number of isoforms for every gene, resulting in >98% sparsity and substantial memory waste. In contrast, our SCHT structure achieves more efficient memory utilization by maintaining variable-sized matrices for each gene, eliminating unnecessary zero-padding. This adaptive structure reduces memory requirements compared to naive tensor approaches while preserving all hierarchical information. Notably, SCHT maintains complete fidelity with filtered isoform matrices, as verified by matched non-zero element counts, demonstrating that our compression introduces no data loss while achieving substantial computational efficiency.

To evaluate the computational efficiency of ScIsoX, we benchmarked the package on three diverse single-cell long-read datasets, which were used in this study. Performance was measured on a MacBook Pro with Apple M1 Pro chip, 32 GB RAM, running R 4.4.3. Runtime and memory usage were tracked for the two main computational steps: (1) SCHT structure creation via create_scht() and (2) isoform complexity metrics calculation via calculate_isoform_complexity_metrics(). Memory usage was measured as the R heap memory increment during function execution. Detailed performance metrics are presented in Additional file 2: Table S6. Processing times scaled reasonably with dataset size, and memory usage remained modest across all datasets.

### Cell type annotation

All datasets used in this study include cell type annotations acquired through different methodologies. For the murine hematopoietic development dataset, cell type annotations were based on experimentally validated labels [5]. For the brain dataset, we utilized preprocessed data and annotations from Joglekar et al. [6]. In that study, computational preprocessing was performed using Seurat [14], and annotations were generated through manual marker gene identification. This approach identified major brain cell populations including excitatory and inhibitory neurons, oligodendrocytes, astrocytes, microglia, vascular cells, and progenitor populations. For the PBMC dataset, we also performed preprocessing using Seurat [14] and subsequently classified cell types computationally using SingleR [15]. The reference dataset contained well-characterized immune cell signatures that enabled identification of T cells, B cells, NK cells, monocytes, and other PBMC subpopulations.

### Validation of metric robustness to data sparsity

To directly address the challenge that single-cell isoform data is inherently sparse, we performed a comprehensive dropout perturbation analysis to empirically test the robustness of our seven core complexity metrics using the mouse brain dataset featured in our study. We systematically introduced additional random dropouts to the non-zero counts of the brain dataset at increasing rates (from 10% to 50%) to simulate increasingly sparse conditions, with 20 independent iterations per level. The stability of the metrics was evaluated using the overlap coefficient, which measures distributional similarity, and the effect size of the perturbation was quantified using Cliff’s delta. The results, detailed in Additional file 1: Fig. S10, demonstrate exceptional robustness. Even under extreme 50% additional dropout, the mean overlap coefficient across all seven metrics remained high at 0.789, and the corresponding effect sizes remained in the negligible-to-small range, confirming that the ScIsoX framework reliably quantifies complexity patterns even from sparse data.

## Supplementary information

Additional file 1. Supplementary_Figures.pdf: Figs. S1-S10.

Additional file 2. Supplementary_Tables.pdf: Tables S1-S7.

Additional file 3. Supplementary_Note.pdf: Detailed case study of *Irf8* isoform co-expression [22–32].

Additional file 4. QC_Report_Blood_Data.html.

Additional file 5. QC_Report_Brain_Data.html.

Additional file 6. QC_Report_PBMC_Data.html.

## Data Availability

No new datasets were generated during the current study. All datasets analyzed in this study are publicly available. The murine early blood development dataset is available from the Gene Expression Omnibus (GEO) under accession number GSE185555 [16] and also from Zenodo [17] (https://doi.org/10.5281/zenodo.5463924), as described in Wang et al., *Science Advances* [5]. The mouse and human brain development datasets are available from the Knowledge Brain Map at https://knowledge.brain-map.org/data/Z0GBA7V12N4J4NNSUHA/summary (mouse) [18] and https:// knowledge.brain-map.org/data/ASP3B09DZ8PXDUYSHDH/summary (human) [19], as described in Joglekar et al., *Nature Neuroscience* [6]. The human PBMC dataset is available from the PacBio Official database [7] (https://downloads.pacbcloud.com/public/dataset/Kinnex-single-cell-RNA), as described in Al’Khafaji et al., *Nature Biotechnology* [7]. ScIsoX (R package) is publicly available under the MIT License [20] (https://github.com/ThaddeusWu/ScIsoX) and is also archived on Zenodo [21] (https://doi.org/10.5281/zenodo.16569859).
