# Supplementary material for: ScIsoX: a multidimensional framework for measuring isoform-level transcriptomic complexity in single cells
Source: Genome Biol. 2025 Sep 22;26:289. doi: 10.1186/s13059-025-03758-5 (PMC12455757; doi:10.1186/s13059-025-03758-5)
Supplement: Supplementary file 1 — Additional file 1. Supplementary_Figures.pdf: Figs. S1-S10. [file 13059_2025_3758_MOESM1_ESM.pdf]

# Additional File 1: Supplementary Figures

# ScIsoX: a multidimensional framework for measuring isoform-level transcriptomic complexity in single cells


Siyuan Wu[1,2,3] and Ulf Schmitz[1,2,4,*]

[1]Computational Biomedicine Lab, College of Science and Engineering, James Cook University, Townsville, Queensland, Australia

[2]Centre for Tropical Bioinformatics and Molecular Biology, James Cook University, Cairns, Queensland, Australia

[3]School of Mathematics, Monash University, Melbourne, Victoria, Australia

[4]Centenary Institute, The University of Sydney, Camperdown, New South Wales, Australia.

[*]Corresponding author: Ulf.Schmitz@jcu.edu.au

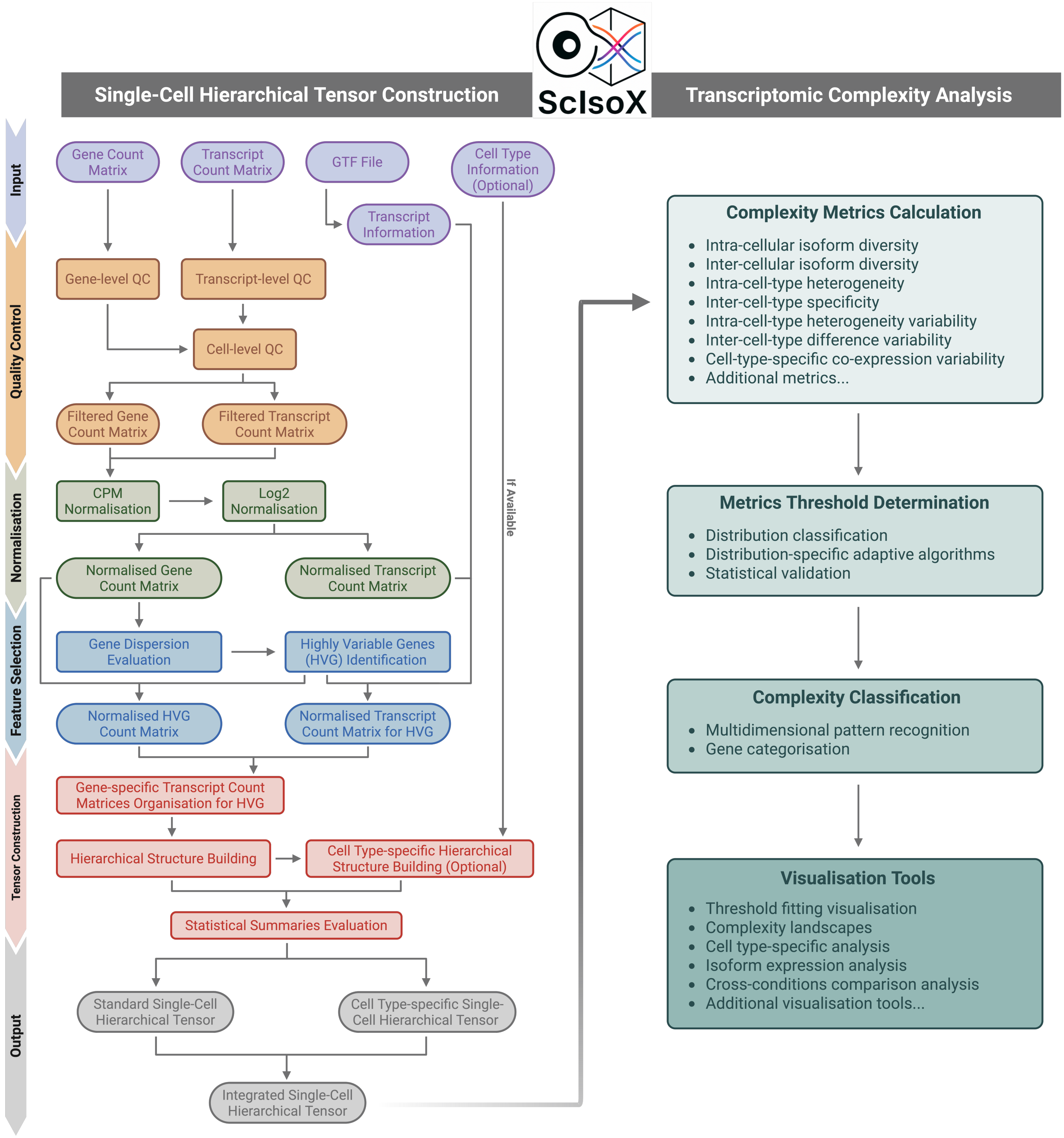


**Fig S1. Detailed workflow of the `ScIsoX` analysis framework.** This is the comprehensive overview of the `ScIsoX` analysis pipeline, beginning with input data on the left (gene count matrix, transcript count matrix, GTF file, and cell type information), proceeding through quality control, normalisation, highly variable genes selection, SCHT construction, and culminating in the various components of transcriptomic complexity analysis on the right. The transcriptomic complexity analysis includes complexity metrics calculation (seven core metrics and additional metrics), distribution-based adaptive threshold determination, multidimensional complexity classification, and rich visualisation tools. Each processing step is clearly defined, forming a complete analytical pipeline that supports extraction of biological meaning from single-cell isoform data. Created with BioRender.com.

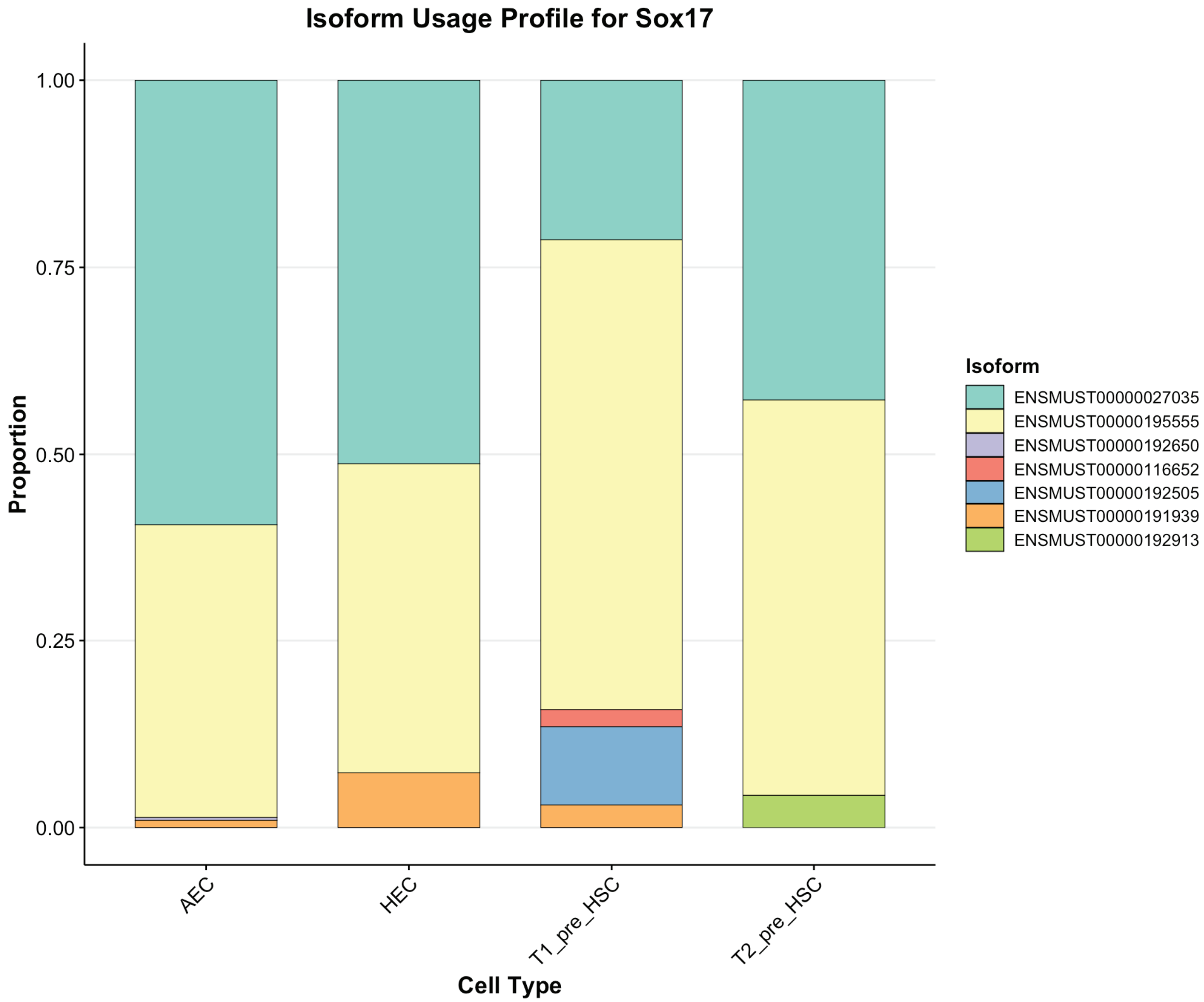


**Fig S2.** ***Sox17*** **isoform usage profiles across different mouse haematopoietic developmental stage.** AEC - arterial endothelial cells; HEC - hemogenic endothelial cells; T1__pre__HSC - Type 1 precursor hematopoietic stem cells; T2__pre__HSC - Type 2 precursor hematopoietic stem cells.

a

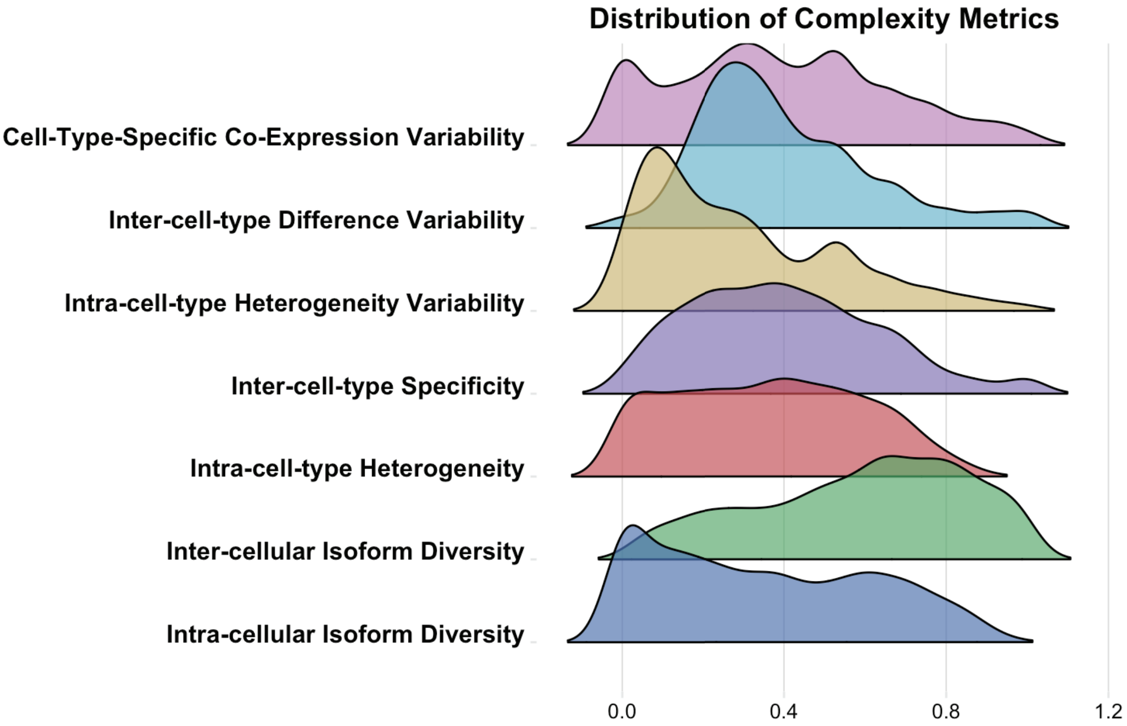

Distribution of Complexity Metrics
Cell-Type-Specific Co-Expression Variability
Inter-cell-type Difference Variability
Intra-cell-type Heterogeneity Variability
Inter-cell-type Specificity
Intra-cell-type Heterogeneity
Inter-cellular Isoform Diversity
Intra-cellular Isoform Diversity
0.0
0.4
0.8
1.2


b

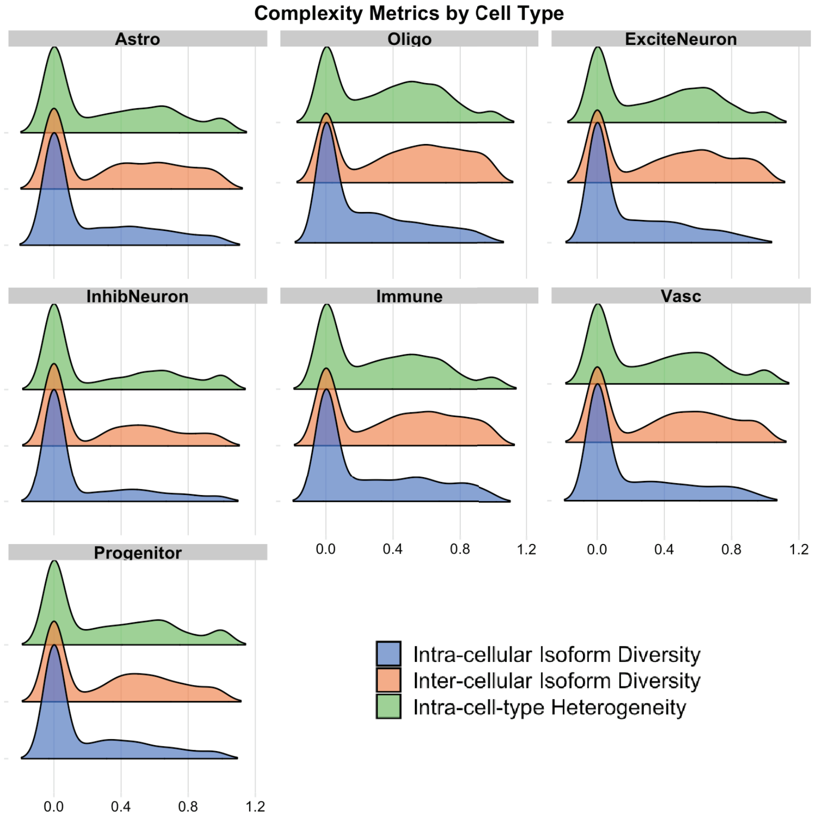

Complexity Metrics by Cell Type
Astro
Oligo
ExciteNeuron
InhibNeuron
Immune
Vasc
Progenitor
0.0
0.4
0.8
1.2
Intra-cellular Isoform Diversity
Inter-cellular Isoform Diversity
Intra-cell-type Heterogeneity

**Fig S3. Distribution characteristics of the seven core complexity metrics and cell-type-specific patterns.** (a) Ridge plots showing distributions of the seven core complexity metrics in the brain dataset, displaying characteristic distributions for each dimension. Each metric exhibits a unique distribution profile, reflecting the biological complexity captured by different dimensions. (b) Comparison of density plots for three key complexity metrics across seven cell types in the brain dataset. Immune - immune cells; Astro - astrocytes; Oligo - oligodendrocytes; ExciteNeuron - excitatory neurons; InhibNeuron - inhibitory neurons; Progenitor - progenitor cells; Vasc - vascular cells.

---

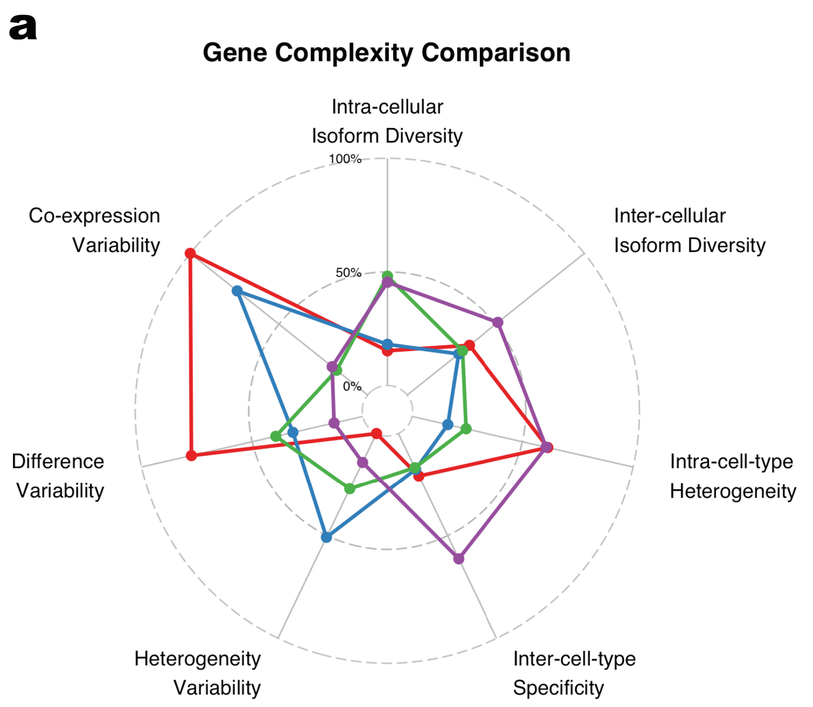
a
Gene Complexity Comparison
Intra-cellular
Isoform Diversity
100%
50%
0%
Inter-cellular
Isoform Diversity
Intra-cell-type
Heterogeneity
Inter-cell-type
Specificity
Heterogeneity
Variability
Difference
Variability
Co-expression
Variability
Gene
Gm2788
Irf8
Pcsk5
Rian

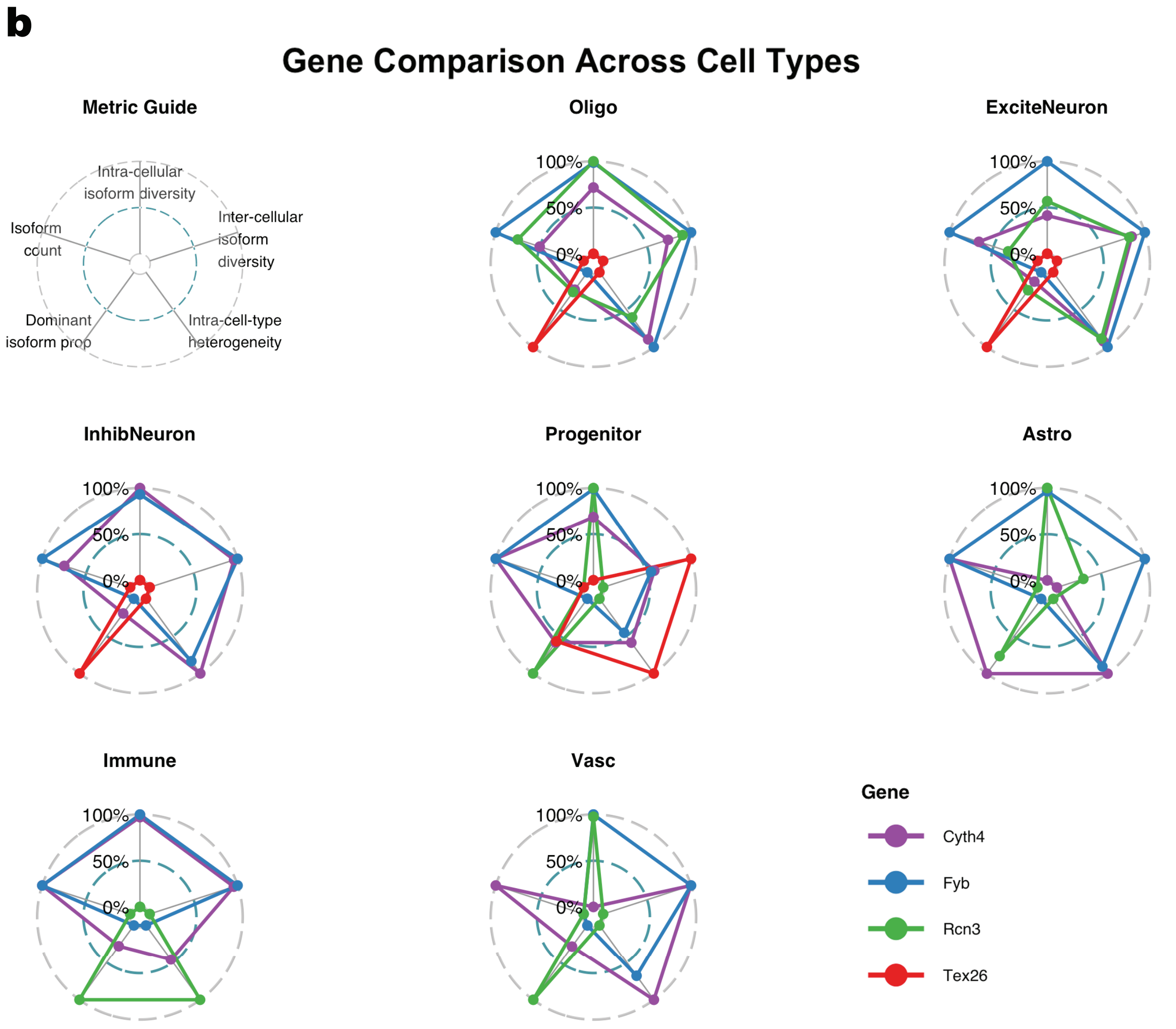
b
Gene Comparison Across Cell Types
Metric Guide
Intra-cellular
isoform diversity
Inter-cellular
isoform
diversity
Intra-cell-type
heterogeneity
Dominant
isoform prop
Isoform
count
Oligo
ExciteNeuron
InhibNeuron
Progenitor
Astro
Immune
Vasc
100%
50%
0%
Gene
Cyth4
Fyb
Rcn3
Tex26

**Fig S4. Radar chart visualisations for multidimensional complexity comparisons.** (a) Seven-dimensional complexity metric comparison radar charts for four genes (*Gm2788*, *Irf8*, *Pcat5*, and *Ran*) in the mouse early blood development dataset, showcasing the unique complexity signatures of different genes. Each gene displays a distinctive complexity fingerprint, reflecting its specific isoform regulatory mechanisms. (b) Complexity profiles of individual genes (*Cyth4*, *Fyb*, *Rcn3*, and *Tex26*) across eight different brain cell types visualised with radar charts, demonstrating cell type specificity of isoform regulation. A metric guide in the top-left corner provides the meaning of the five axes in the radar charts. This visualisation method effectively captures and compares high-dimensional complexity data. Immune - immune cells; Astro - astrocytes; Oligo - oligodendrocytes; ExciteNeuron - excitatory neurons; InhibNeuron - inhibitory neurons; Progenitor - progenitor cells; Vasc - vascular cells.

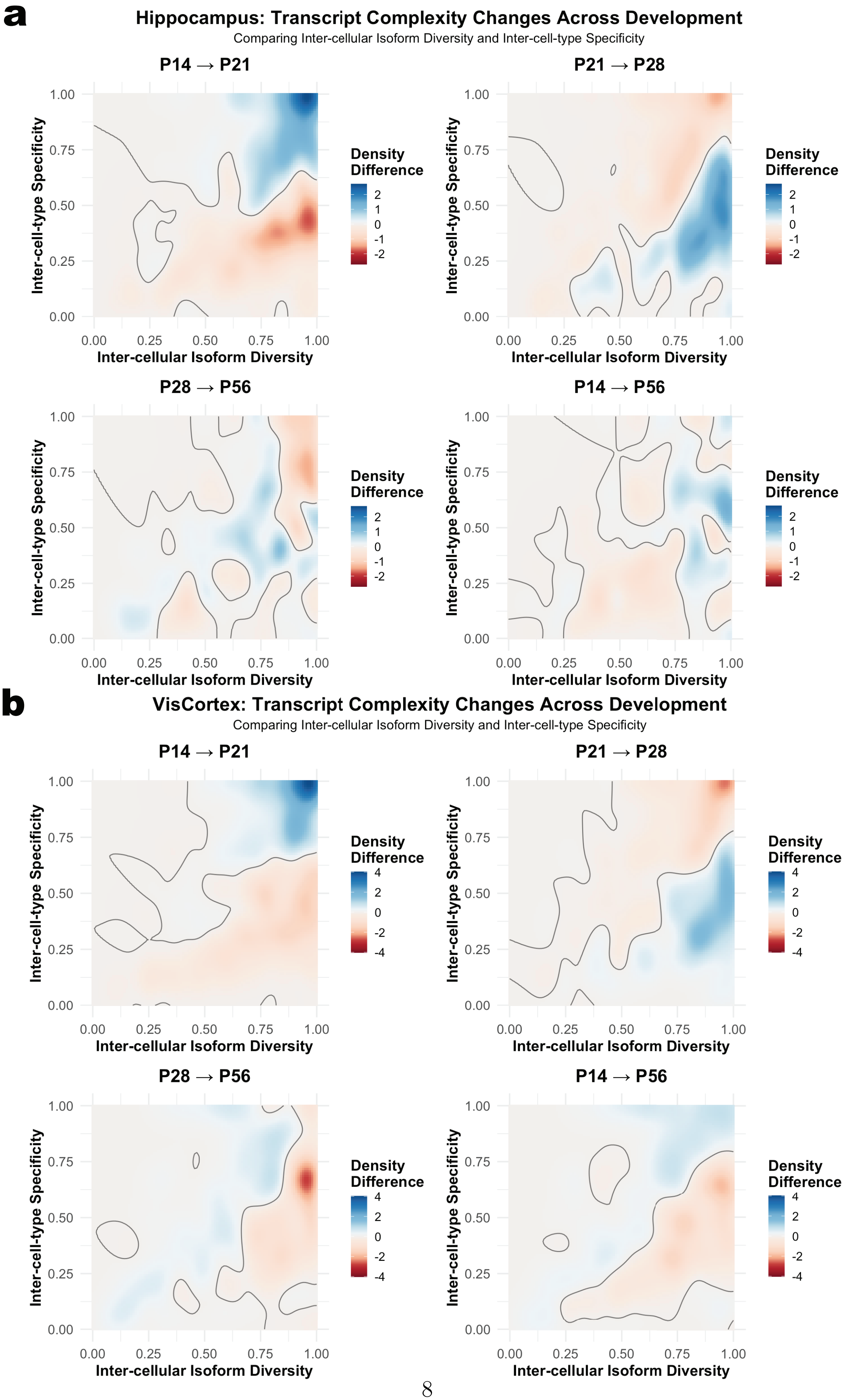
a
Hippocampus: Transcript Complexity Changes Across Development
Comparing Inter-cellular Isoform Diversity and Inter-cell-type Specificity
P14 → P21
P21 → P28
P28 → P56
P14 → P56
Inter-cell-type Specificity
Inter-cellular Isoform Diversity
0.00
0.25
0.50
0.75
1.00
Density Difference
2
1
0
-1
-2
b
VisCortex: Transcript Complexity Changes Across Development
Comparing Inter-cellular Isoform Diversity and Inter-cell-type Specificity
P14 → P21
P21 → P28
P28 → P56
P14 → P56
Inter-cell-type Specificity
Inter-cellular Isoform Diversity
0.00
0.25
0.50
0.75
1.00
Density Difference
4
2
0
-2
-4

**Fig S5. Dynamic changes in transcriptomic complexity across postnatal developmental stages in mouse hippocampus and visual cortex regions.** (a) Density difference maps comparing inter-cellular isoform diversity and inter-cell-type specificity in mouse hippocampus across four developmental stages (Days 14, 21, 28 and 56). Red regions indicate gene decreasing density while blue regions indicate increasing density. Each transition period shows a unique pattern of changes. (b) Complexity change patterns in mouse visual cortex across four developmental stages, showing region-specific differences compared to hippocampus. Colour scales indicate the intensity of density differences. This analysis reveals spatiotemporal changes in transcriptomic complexity during brain development.

---

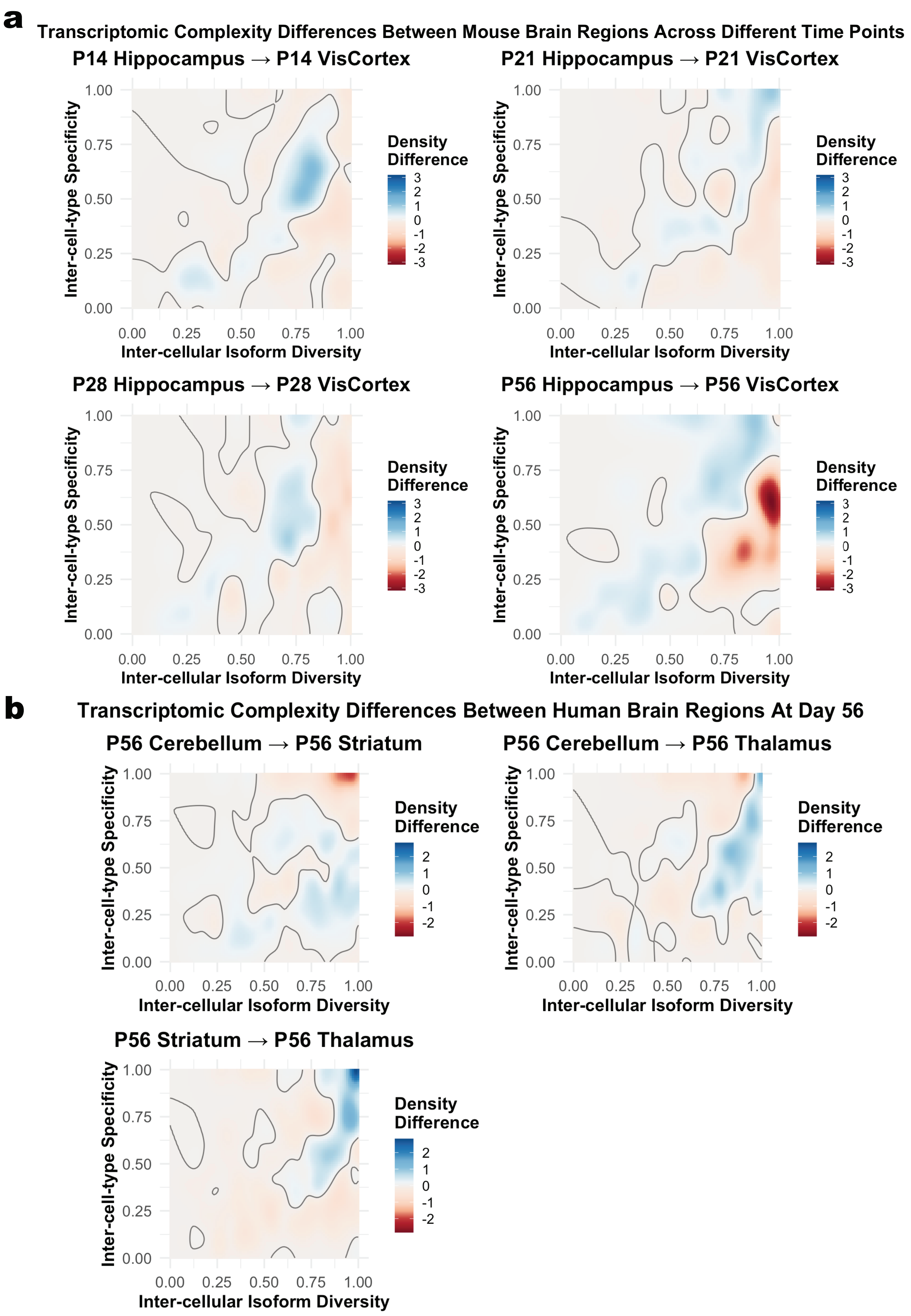
a
Transcriptomic Complexity Differences Between Mouse Brain Regions Across Different Time Points
P14 Hippocampus → P14 VisCortex
P21 Hippocampus → P21 VisCortex
P28 Hippocampus → P28 VisCortex
P56 Hippocampus → P56 VisCortex
Inter-cell-type Specificity
Inter-cellular Isoform Diversity
0.00
0.25
0.50
0.75
1.00
Density Difference
3
2
1
0
-1
-2
-3
b
Transcriptomic Complexity Differences Between Human Brain Regions At Day 56
P56 Cerebellum → P56 Striatum
P56 Cerebellum → P56 Thalamus
P56 Striatum → P56 Thalamus
Inter-cell-type Specificity
Inter-cellular Isoform Diversity
Density Difference
2
1
0
-1
-2

**Fig S6. Transcript complexity differences between mouse brain regions across postnatal developmental stages and between adult human brain regions.** (a) Developmental changes in transcript complexity profiles comparing hippocampus and visual cortex regions across different time points in mouse brain (Days 14, 21, 28 and 56). Each density difference map displays changes in the relationship between inter-cellular isoform diversity and inter-cell-type specificity, with blue indicating regions of increased gene density and red indicating decreased density. These developmental comparisons reveal dynamic shifts in transcriptomic complexity patterns as brain circuits mature. (b) Transcript complexity differences between adult human brain regions. The figure presents density difference maps between three pairs of distinct brain regions (Cerebellum-Striatum, Cerebellum-Thalamus, and Striatum-Thalamus). Each comparison displays regional differences in inter-cellular isoform diversity and inter-cell-type specificity, with blue indicating greater gene density in the first region and red indicating greater density in the second region.

---

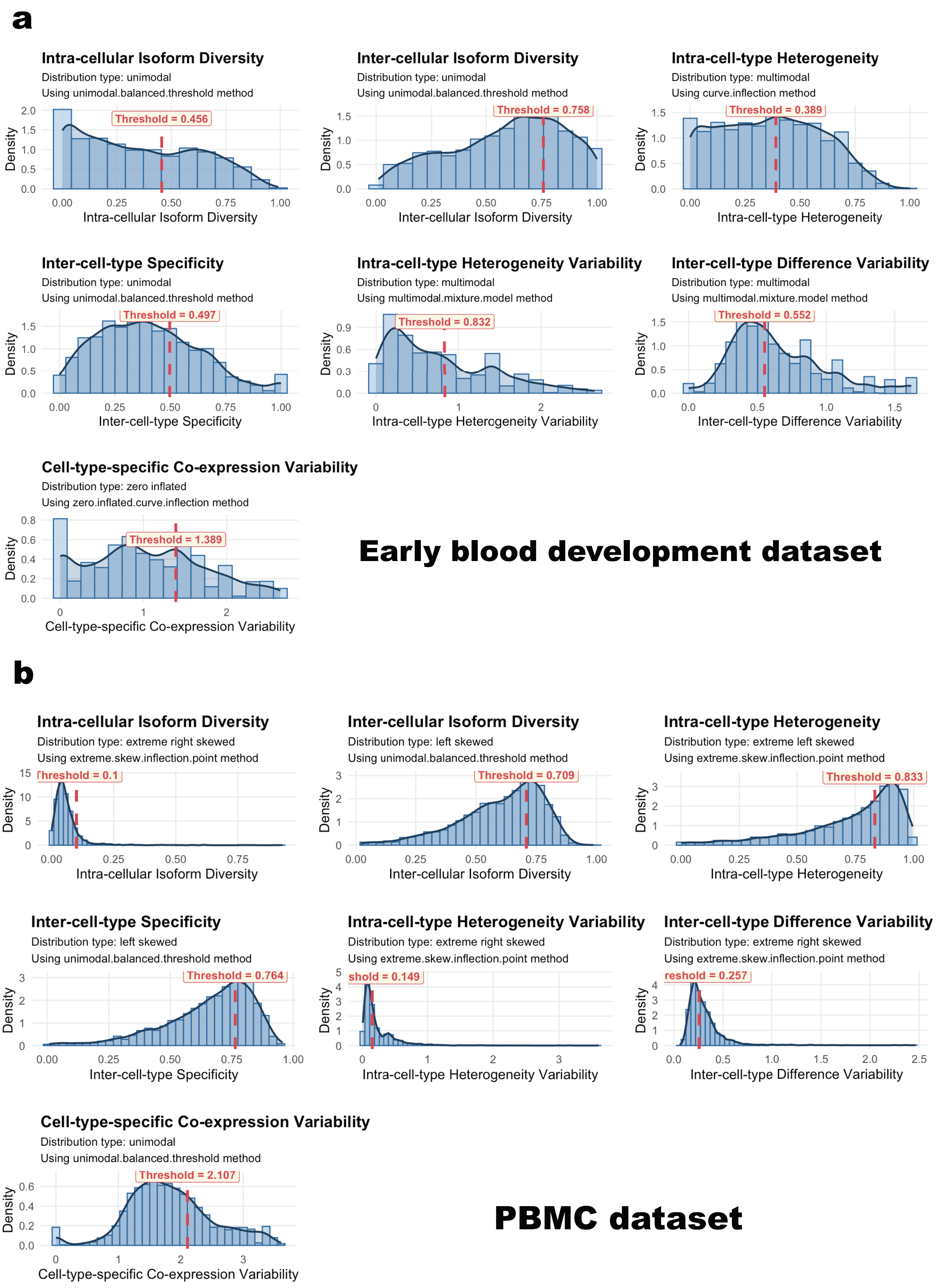


**Fig S7. Visualisation of complexity metrics threshold determination for early blood development and PBMC datasets.**

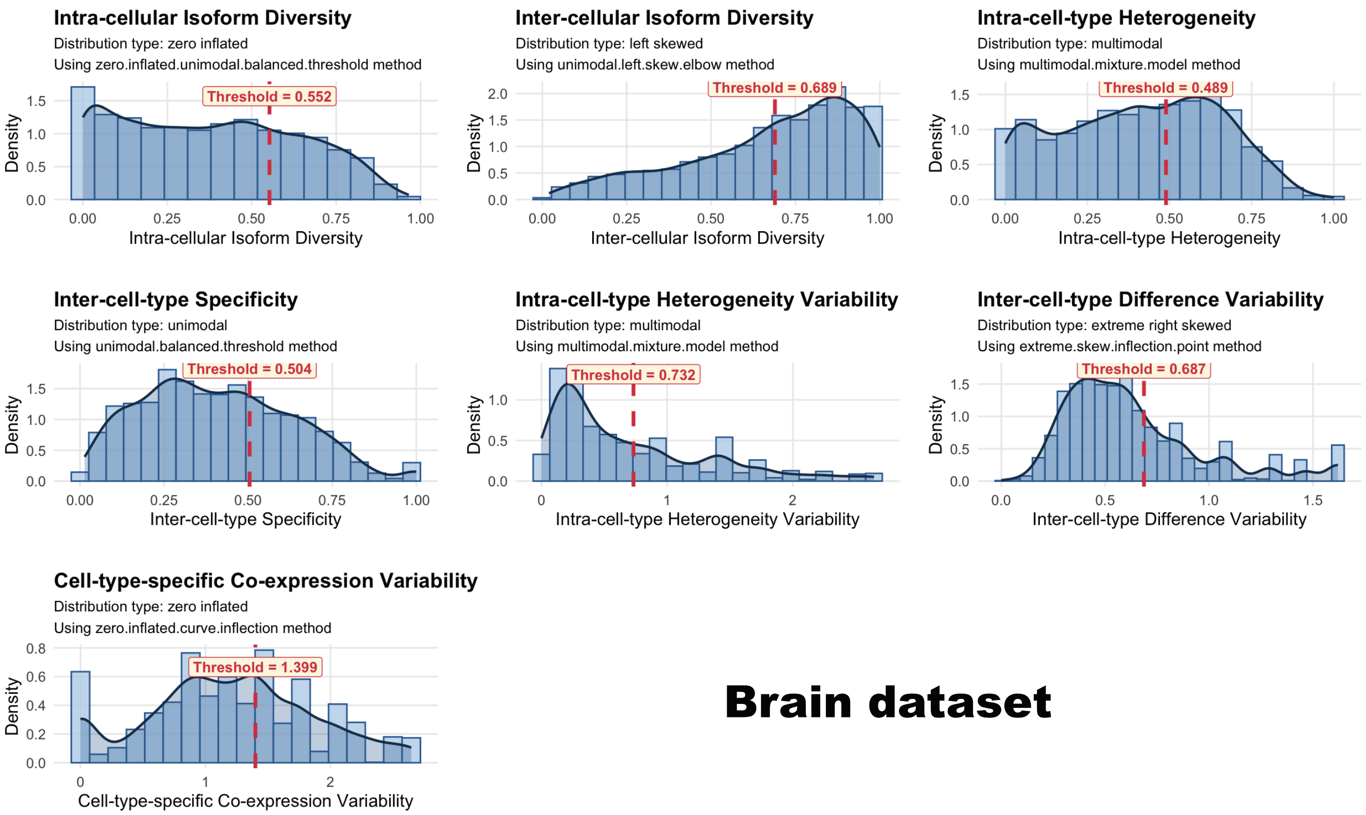


**Fig S8. Visualisation of complexity metrics threshold determination for brain dataset.**

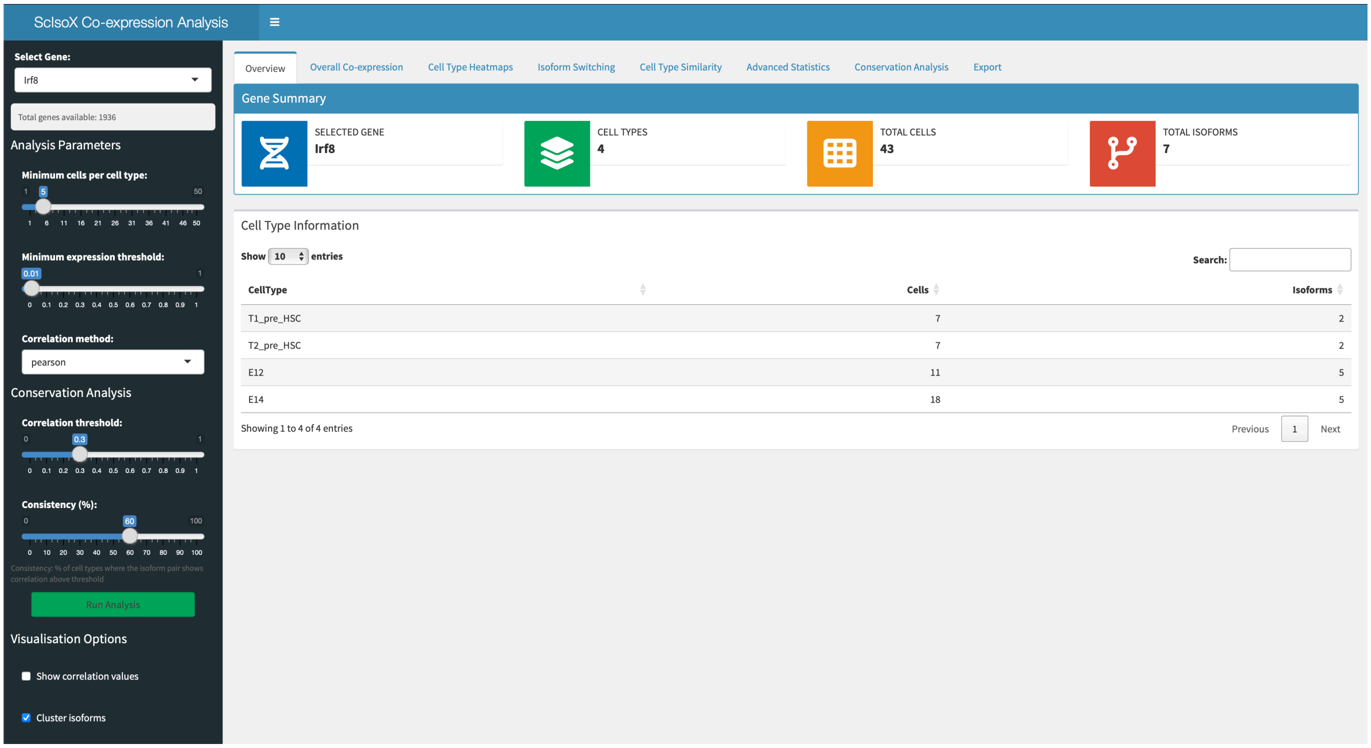


**Fig S9. Interactive exploration of *Irf8* co-expression using the `ScIsoX` co-expression analysis Shiny app.** The graphical user interface enables the selection of specific genes (e.g., *Irf8*) to visualise co-expression heatmaps, perform statistical validation and analyse conservation patterns across cell types.

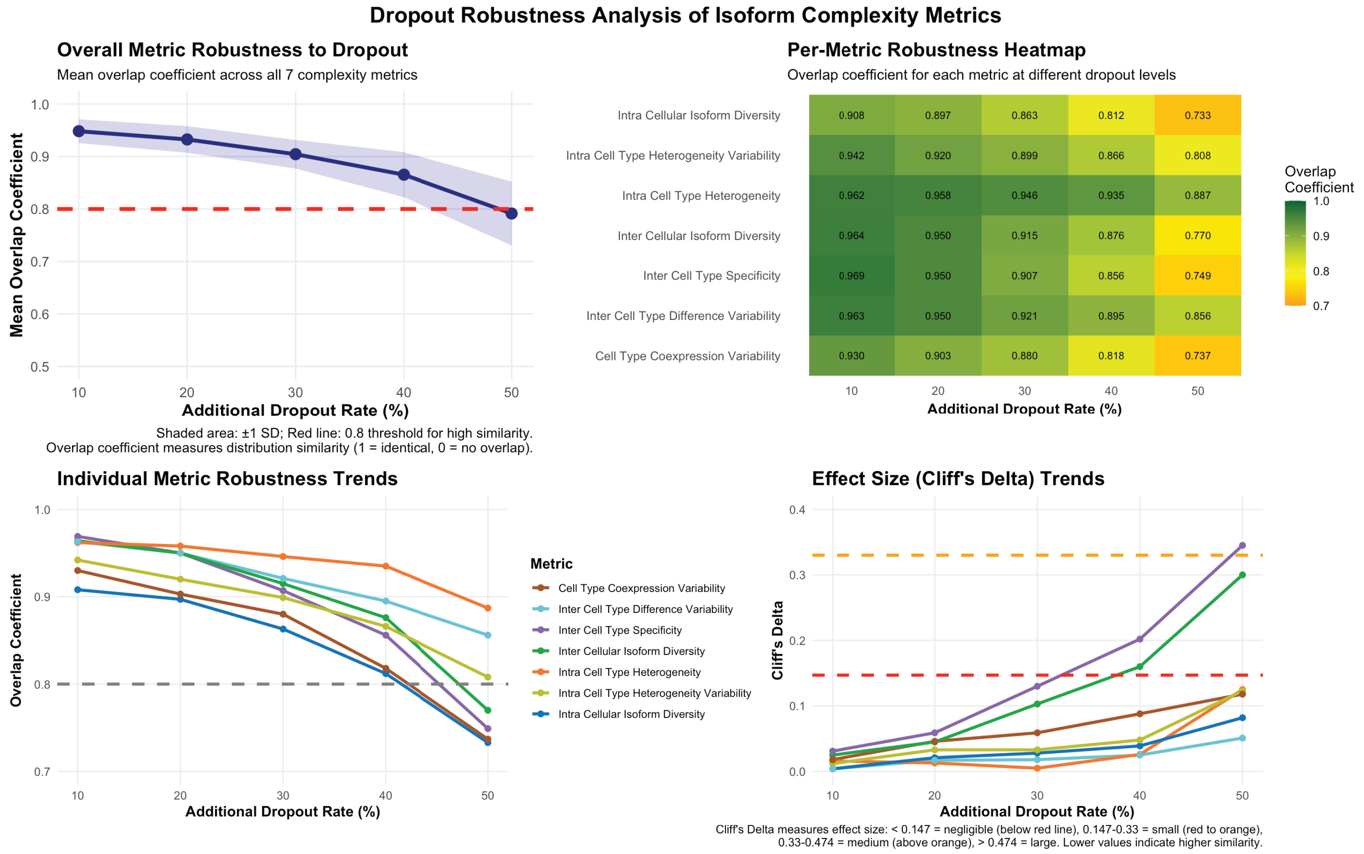


**Fig S10. Robustness of `ScIsoX` complexity metrics to data sparsity evaluated by dropout perturbation analysis.** To empirically test the stability of the seven core complexity metrics, we performed a simulation by systematically introducing additional random dropout (from 10% to 50%) to the non-zero counts of the brain dataset over 20 independent iterations. **(Top-left)** The overall robustness across all seven metrics, measured by the mean overlap coefficient. The overlap coefficient measures the similarity of the metric distributions before and after dropout (1 = identical, 0 = no overlap). The red dashed line at 0.8 indicates a threshold for high similarity. The shaded area represents ±1 standard deviation across iterations. **(Top-right)** A per-metric heatmap showing the overlap coefficient for each of the seven metrics at each dropout level, demonstrating high stability for all metrics even at high dropout rates. **(Bottom-left)** Individual trend lines showing the overlap coefficient for each metric as a function of increasing dropout rate. **(Bottom-right)** The effect size of the perturbation, measured using Cliff's Delta. Values remain in the negligible ($< 0.147$) to small (0.147-0.33) range across all tested conditions, confirming that increasing sparsity does not dramatically alter the metric calculations.
