# Supplementary material for: ScIsoX: a multidimensional framework for measuring isoform-level transcriptomic complexity in single cells
Source: Genome Biol. 2025 Sep 22;26:289. doi: 10.1186/s13059-025-03758-5 (PMC12455757; doi:10.1186/s13059-025-03758-5)
Supplement: Supplementary file 2 — Additional file 2. Supplementary_Tables.pdf: Tables S1-S7. [file 13059_2025_3758_MOESM2_ESM.pdf]

Additional File 2: Supplementary Tables

ScIsoX: a multidimensional framework for measuring isoform-level transcriptomic complexity in single cells

Siyuan Wu[1,2,3] and Ulf Schmitz[1,2,4,*]

[1]Computational Biomedicine Lab, College of Science and Engineering, James Cook University, Townsville, Queensland, Australia

[2]Centre for Tropical Bioinformatics and Molecular Biology, James Cook University, Cairns, Queensland, Australia

[3]School of Mathematics, Monash University, Melbourne, Victoria, Australia

[4]Centenary Institute, The University of Sydney, Camperdown, New South Wales, Australia.

[*]Corresponding author: Ulf.Schmitz@jcu.edu.au

Metric	Description	Calculation Method	Biological Interpretation	High Values Indicate	Low Values Indicate
**Intra-cellular Isoform Diversity**	Measures the tendency for a gene to co-express multiple isoforms within individual cells	Weighted average of per-cell normalised Shannon entropy	Reveals whether genes express multiple isoforms simultaneously	Active co-expression of multiple isoforms within individual cells	Expression of predominantly one isoform per cell
**Inter-cellular Isoform Diversity**	Quantifies the diversity of isoforms expressed by a gene across the whole cell population	Normalised Shannon entropy of average isoform expression	Shows the overall diversity of isoforms expressed in the tissue	Multiple isoforms present across the cell population	One or few dominant isoforms across the population
**Intra-cell-type Heterogeneity**	Measures cell-to-cell variation in isoform usage within each cell type	Average Jensen-Shannon distance between cells within a cell type	Reveals whether cells of the same type use isoforms consistently	High cell-to-cell variability in isoform usage	Consistent isoform usage pattern within the cell type
**Inter-cell-type Specificity**	Quantifies how differently a gene uses its isoforms across different cell types	Average Jensen-Shannon distance between cell type-specific isoform profiles	Shows whether isoform usage is cell-type-specific	Cell types use distinct isoform repertoires	Similar isoform usage across cell types
**Intra-cell-type Heterogeneity Variability**	Measures whether certain cell types show particularly high cellular heterogeneity	Coefficient of variation of heterogeneity values across cell types	Reveals if heterogeneity is concentrated in specific cell types	Some cell types have much higher internal heterogeneity than others	Consistent levels of heterogeneity across all cell types
**Inter-cell-type Difference Variability**	Measures whether certain cell type pairs show particularly significant differences	Coefficient of variation of pairwise Jensen-Shannon distances	Shows whether differences are concentrated between specific cell type pairs	Certain cell type pairs have dramatically different isoform usage	Relatively uniform differences between all cell type pairs
**Cell-type-specific Co-expression Variability**	Measures whether a gene exhibits different co-expression patterns in different cell types	Coefficient of variation of mean intra-cellular diversity across cell types	Reveals if co-expression patterns vary by context	Gene exhibits different co-expression patterns in different cell types	Consistent co-expression mechanism across all cell types

Table S1. Core Transcriptomic Complexity Metrics.

Metric	Description	Calculation Method	Biological Interpretation
**IDI Difference**	The difference between inter-cellular and intra-cellular isoform diversity	Inter-cellular isoform diversity – Intra-cellular isoform diversity	Positive values indicate greater diversity across cells than within cells, suggesting cell specialisation; negative values suggest cells co-express many isoforms but in similar patterns
**Simpson Index**	Alternative diversity measure more sensitive to dominant isoforms	1 - sum(iso_props^2)	Accounts for the probability that two randomly selected transcripts belong to different isoforms; complements Shannon entropy-based metrics
**Evenness**	Normalised diversity controlling for number of expressed isoforms	inter_cellular_isoform_diversity / log2(n_expressed_isoforms)	Measures how equally expressed the isoforms are; values near 1 indicate equal expression, near 0 indicate dominance by few isoforms
**Dominant Isoform Proportion**	The proportion of the most abundant isoform	max(iso_props)	Indicates the degree of dominance by a single isoform; high values suggest one functional isoform dominates
**Number of Expressed Isoforms**	Count of isoforms with detectable expression	sum(iso_means > 0)	Indicates the absolute diversity of isoforms being expressed; reflects the gene's splicing complexity
**Percentage of Multi-isoform Cells**	Percentage of cells expressing multiple isoforms of a given gene	(Multi-isoform cell count / cells expressing) × 100	Direct measure of co-expression at the single-cell level; high values indicate widespread co-expression
**Cells Expressing**	Number of cells with detectable expression of a given gene	sum(cell_sums > 0)	Indicates the prevalence of the gene's expression across the cell population
**Percentage of Cells Expressing**	Percentage of total cells expressing the gene	(cells_expressing / total_cells) × 100	Measures how widespread the gene's expression is; distinguishes ubiquitous from restricted expression patterns

Table S2. Additional Transcriptomic Complexity Metrics.

Dimension	High Classification	Low Classification	Interpretation
**Intra-cellular Isoform Diversity**	High Isoform Co-expression	Low Isoform Co-expression	Distinguishes genes based on single-cell co-expression patterns
**Inter-cellular Isoform Diversity**	High Isoform Diversity	Low Isoform Diversity	Distinguishes genes based on population-level isoform diversity
**Intra-cell-type Heterogeneity**	High Cellular Heterogeneity	Low Cellular Heterogeneity	Distinguishes genes based on cell-to-cell variability within cell types
**Inter-cell-type Specificity**	Cell-Type-Specific Isoform Expression	Cell-Type-Independent Isoform Expression	Distinguishes genes based on cell type specialisation of isoform usage
**Intra-cell-type Heterogeneity Variability**	Variable Heterogeneity Across Cell Types	Consistent Heterogeneity Across Cell Types	Distinguishes genes based on targeted vs. uniform heterogeneity
**Inter-cell-type Difference Variability**	High Cell-Type Distinctions	Low Cell-Type Distinctions	Distinguishes genes based on focused vs. gradual differentiation
**Cell-type-specific Co-expression Variability**	Cell-Type-Adaptive Co-expression	Cell-Type-Consistent Co-expression	Distinguishes genes based on context-dependent isoform co-expression

Table S3. Metrics Classification System.

Pattern	Biological Significance	Example Genes	Potential Functional Implications
**High Intra-cellular Isoform Diversity + High Inter-cellular Isoform Diversity**	Rich isoform landscape both within and across cells	Genes involved in complex cellular processes	Multiple functional isoforms with complementary roles; high regulatory complexity
**Low Intra-cellular Isoform Diversity + High Inter-cellular Isoform Diversity**	Cell specialisation in isoform usage	Cell type marker genes, specialised receptors	Cell type-specific isoform selection; potential for specialised functions
**Low Intra-cellular Isoform Diversity + Low Inter-cellular Isoform Diversity**	Single dominant isoform usage	Housekeeping genes, core cellular machinery	Conserved function requiring specific isoform; limited need for diversity
**High Intra-cellular Isoform Diversity + Low Inter-cellular Isoform Diversity**	Consistent co-expression of specific isoform sets	Genes requiring balanced isoform ratios	Functional requirement for multiple isoforms within same cell; potential isoform cooperation
**High Cell-type Specificity + Low Difference Variability**	Consistent differentiation across all cell types	Lineage-specific transcription factors	Gradual divergence in isoform usage corresponding to cellular differentiation
**High Cell-type Specificity + High Difference Variability**	Targeted differentiation between specific lineages	Immune recognition molecules, neuronal connectivity factors	Sharp transitions in isoform usage between specific lineages; potential for dramatic functional shifts

Table S4. Example of Complexity Patterns.

Complexity Metric	NA Generation Condition	Biological Interpretation	Classification Label	Mathematical Rationale
**Intra-cellular Isoform Diversity**	Gene expresses only one isoform across all cells	No alternative splicing detected; single isoform dominance	"Unclassified"	Shannon entropy undefined when only one category exists; weighted average cannot capture diversity
**Inter-cellular Isoform Diversity**	Only one isoform expressed across cell population	Population-wide isoform preference; no isoform diversity	"Unclassified"	Normalised Shannon entropy approaches 0; $\log_2(1) = 0$ creates mathematical boundary condition
**Intra-cell-type Heterogeneity**	< 3 cells per cell type express the gene	Insufficient sampling for robust heterogeneity calculation	"Unclassified"	Jensen-Shannon distance requires ≥ 2 cells for pairwise comparisons; statistical robustness needs ≥ 3 cells
**Inter-cell-type Specificity**	Gene expressed in 0 qualifying cell types (no cell type meets minimum data requirements)	Complete absence of qualifying cell type data	"Single-Cell Type Expression"	Special boundary handling: Returns 1.0 for exactly 1 cell type (maximum specificity), NA only when no cell types qualify
**Intra-cell-type Heterogeneity Variability**	< 2 cell types with sufficient heterogeneity data	Cannot assess heterogeneity variation across cell types	"Insufficient Cell Type Data"	Coefficient of variation requires ≥ 2 cell types with calculable heterogeneity values
**Inter-cell-type Difference Variability**	Insufficient pairwise cell type comparisons available	Cannot assess variation in cell type distinctiveness	"Insufficient Difference Data"	Requires multiple pairwise Jensen-Shannon distances to calculate variability statistics
**Cell-type-specific Co-expression Variability**	< 2 cell types with quantifiable co-expression data	Cannot assess co-expression pattern variation across contexts	"Insufficient Data"	Coefficient of variation of mean intra-cellular diversity requires ≥ 2 cell types with IDI measurements

Table S5. Explanation of NA values.

Data Structure	Blood Data	Brain Data	PBMC Data
Original Transcript Matrix			
Number of genes	55,487	31,335	27,176
Number of isoforms	142,238	132,119	792,617
Number of cells	205	301	12,852
Non-zero elements	2,457,789	5,598,505	13,127,059
Zero elements	26,701,001	34,169,314	10,173,586,625
Total elements	29,158,790	39,767,819	10,186,713,684
Sparsity (%)	91.57	85.92	99.87
Filtered Transcript Matrix (Post-QC HVG)			
Number of genes	1,936	2,248	2,980
Number of isoforms	7,327	8,637	167,204
Number of cells	181	272	11,640
Non-zero elements	73,057	152,738	1,356,892
Zero elements	1,253,130	2,196,526	1,944,897,668
Total elements	1,326,187	2,349,264	1,946,254,560
Sparsity (%)	94.49	93.50	99.93
Naive 3D Tensor (Post-QC HVG)			
Required dimensions	1,936×21×181	2,248×16×272	2,980×268×11,640
Non-zero elements	73,057	152,738	1,356,892
Zero elements	7,285,679	9,630,558	9,294,812,708
Total elements	7,358,736	9,783,296	9,296,169,600
Sparsity (%)	99.01	98.44	99.99
SCHT Structure (Post-QC HVG)			
Non-zero elements	73,057	152,738	1,356,892
Zero elements	154,780	295,087	78,499,452
Total elements	227,837	447,825	79,856,344
Sparsity (%)	67.93	65.89	98.30
Zero Elements Avoided by SCHT			
vs Original Matrix	26,546,221	33,874,227	10,095,087,173
vs Filtered Matrix	1,098,350	1,901,439	1,866,398,216
vs Naive 3D Tensor	7,130,899	9,335,471	9,216,313,256

Table S6. Memory efficiency comparison: SCHT versus alternative data structures. QC: quality control; HVG: highly variable gene.

Metric	Blood Data	Brain Data	PBMC Data
Dataset characteristics			
Number of genes	55,487	31,335	27,176
Number of transcripts	142,238	132,119	792,617
Number of cells	205	395	12,852
Number of cell types	7	7	26
Median genes per cell	7,368	10,072	814
Processing time (seconds)			
SCHT creation	34.77	47.9	693.9
Complexity metrics calculation	113.1	130.0	1,804.1
Total processing time	147.9	177.9	2,498.0
Memory utilised (incremental) (MB)			
SCHT creation	181.0	466.5	2,118.3
Complexity metrics calculation	663.2	866.5	2,416.6

Table S7. `ScIsoX` computational performance across diverse single-cell long-read datasets. Performance was measured on a MacBook Pro with Apple M1 Pro chip, 32GB RAM, running `R 4.4.3`.
