# Supplementary material for: ScIsoX: a multidimensional framework for measuring isoform-level transcriptomic complexity in single cells
Source: Genome Biol. 2025 Sep 22;26:289. doi: 10.1186/s13059-025-03758-5 (PMC12455757; doi:10.1186/s13059-025-03758-5)
Supplement: Supplementary file 3 — Additional file 3. Supplementary_Note.pdf: Detailed case study of Irf8 isoform co-expression [22–32]. [file 13059_2025_3758_MOESM3_ESM.pdf]

Additional File 3: Supplementary Note

ScIsoX: a multidimensional framework for measuring isoform-level transcriptomic complexity in single cells

Siyuan Wu[1,2,3] and Ulf Schmitz[1,2,4,*]

[1]Computational Biomedicine Lab, College of Science and Engineering, James Cook University, Townsville, Queensland, Australia

[2]Centre for Tropical Bioinformatics and Molecular Biology, James Cook University, Cairns, Queensland, Australia

[3]School of Mathematics, Monash University, Melbourne, Victoria, Australia

[4]Centenary Institute, The University of Sydney, Camperdown, New South Wales, Australia.

[*]Corresponding author: Ulf.Schmitz@jcu.edu.au

A case study on the co-expression dynamics of *Irf8* isoforms

Abstract

This note details a case study on the master transcription factor Interferon Regulatory Factor 8 (*Irf8*) within the context of mouse early blood development, conducted using the interactive Shiny application provided by the `ScIsoX` package. The analysis revealed two distinct co-expression patterns. The first pattern demonstrates a strong, stage-specific positive correlation between a protein-coding isoform and an intron-retained variant, exclusively at the E12 embryonic stage. This relationship, while highly statistically significant (FDR q-value < 0.001), showed instability in bootstrap analysis, correctly suggesting its context-specific nature. The second pattern involves a canonical, full-length protein-coding isoform and a shorter, alternative isoform, which exhibit a dynamically mixed correlation (either positive or negative) across different haematopoietic development stages. This case study deconstructs these two patterns by integrating our software's analytical outputs with annotations from public databases. The findings suggest that these patterns are not stochastic noise but rather represent two distinct and well-documented paradigms of dynamic gene regulation: post-transcriptional buffering and functional fine-tuning via protein isoform switching.

Analysis Workflow and Findings

The entire analysis was performed using the `ScIsoX` co-expression analysis Shiny application, which allows for a multi-step, interactive investigation of isoform relationships. The workflow began with statistical validation of overall correlations, followed by conservation analysis to identify context-dependent patterns, and concluded with inspection of cell-type-specific heatmaps and isoform transition plots.

Annotation of Key *Irf8* Isoforms

To provide a data-driven foundation for the analysis, the structural and functional annotations for the four key mouse *Irf8* transcripts were consolidated from the Ensembl Genome Databases (Table SN1). This table translates the Ensembl transcript identifiers into concrete biological entities, highlighting the fundamental structural differences that form the basis for the subsequent functional hypotheses.

Table SN1. Annotation of Key *Irf8* Isoforms discussed in this case study.

Ensembl Transcript ID	Transcript Name	Biotype	Protein Product
ENSMUST00000162001	Irf8-205	Protein coding	424 aa (Full-length)
ENSMUST00000160388	Irf8-202	Retained intron	No protein
ENSMUST00000047737	Irf8-201	Protein coding	424 aa (Full-length)
ENSMUST00000160943	Irf8-204	Protein coding	Truncated protein

Step 1: Statistical Validation Reveals Contrasting Patterns

The initial exploration began in the "Advanced Statistics" tab of the Shiny application. A confidence interval analysis of the overall co-expression correlations for *Irf8* across all cell types identified the isoform pair `ENSMUST00000160388-ENSMUST00000162001` as having one of the highest and statistically significant positive correlations (overall correlation $r = 0.899$, FDR q-value < 0.001)[1] (Fig. SN1a-1b). Interestingly, a bootstrap stability analysis revealed this same pair to be highly unstable (Bootstrap SD = 0.451) (Fig. SN1c). This combination of high overall correlation but high bootstrap instability strongly suggested that the co-expression was not consistent across all cell types, but was likely driven by a powerful effect in a specific context.

Step 2: Conservation Analysis Pinpoints Context-Specific Pairs

To investigate this hypothesis, we used the "Conservation Analysis" module. This analysis confirmed that the `...160388-...162001` pair has a cell-type-specific co-expression pattern. This module also highlighted another interesting pair, `ENSMUST00000047737-ENSMUST00000160943`, for its "mixed" correlation pattern across different stages. These two pairs were selected for deeper investigation.

Step 3: Deconstruction of Co-expression Patterns

Case 1: The Stage-Specific Protein-Coding/Non-Coding Pair

Subsequent inspection of cell-type-specific visualisations confirmed the stage-specific nature of the `...160388-...162001` pair. The isoform usage transition plot shows that the proportional expression of both transcripts synchronously peaks at the E12 embryonic stage (Fig.

[1]Note that this overall correlation includes all cells in the dataset, whereas the conservation analysis examines correlations within specific cell types where both isoforms are expressed, hence the difference in correlation values.

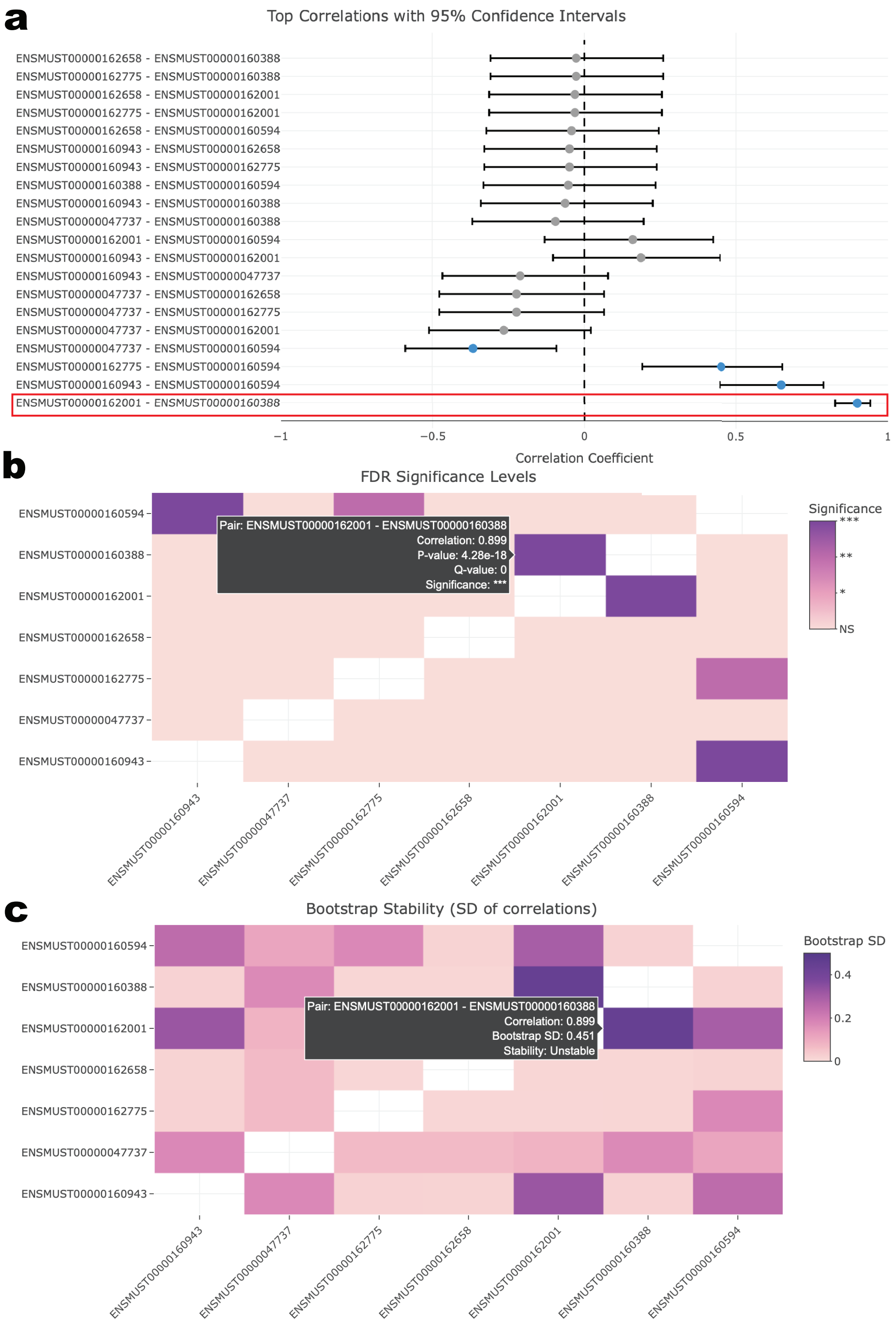

Figure SN1. Statistical validation of *Irf8* isoform co-expression patterns. (a) Forest plot showing isoform pair correlations with 95% confidence intervals. The ENSMUST00000162001-ENSMUST00000160388 pair (highlighted in red box) exhibits the highest positive correlation ($r = 0.899$). (b) Heatmap of FDR-corrected significance levels for isoform pair correlations. The highlighted pair shows highly significant co-expression (q-value < 0.001). (c) Bootstrap stability analysis revealing high instability ($SD = 0.451$) for the ENSMUST00000162001-ENSMUST00000160388 pair, suggesting context-specific rather than constitutive co-expression.

Conserved Isoform Pairs

Show 10 **entries** **Search:**

isoform_pair	mean_correlation	conservation_pattern	n_cell_types	consistency
ENSMUST00000162001-ENSMUST00000160388	0.922	Cell_Type_Specific	1	1.000
ENSMUST00000047737-ENSMUST00000162001	-0.475	Conserved_Negative	2	1.000
ENSMUST00000160943-ENSMUST00000162001	0.465	Non_Conserved	2	0.500
ENSMUST00000162001-ENSMUST00000160594	0.428	Non_Conserved	2	0.500
ENSMUST00000160943-ENSMUST00000160594	0.383	Non_Conserved	2	0.500
ENSMUST00000047737-ENSMUST00000162658	-0.341	Cell_Type_Specific	1	1.000
ENSMUST00000160943-ENSMUST00000047737	-0.247	Mixed	4	1.000
ENSMUST00000047737-ENSMUST00000160388	-0.234	Cell_Type_Specific	1	0.000
ENSMUST00000047737-ENSMUST00000160594	-0.161	Non_Conserved	2	0.500
ENSMUST00000160943-ENSMUST00000160388	-0.131	Cell_Type_Specific	1	0.000

Showing 1 to 10 of 14 entries Previous 1 2 Next

Figure SN2. Conservation analysis of *Irf8* isoform co-expression patterns across cell types. The table displays isoform pairs categorised by their conservation patterns: Cell_Type_Specific (consistent correlation in specific cell types only), Conserved_Negative (consistent negative correlation across multiple cell types), Conserved_Positive (consistent positive correlation across multiple cell types), and Mixed (alternating positive and negative correlations). Two pairs are highlighted for detailed analysis: ENSMUST00000162001-ENSMUST00000160388 showing cell-type-specific pattern, and ENSMUST00000160943-ENSMUST00000047737 exhibiting mixed correlation patterns.

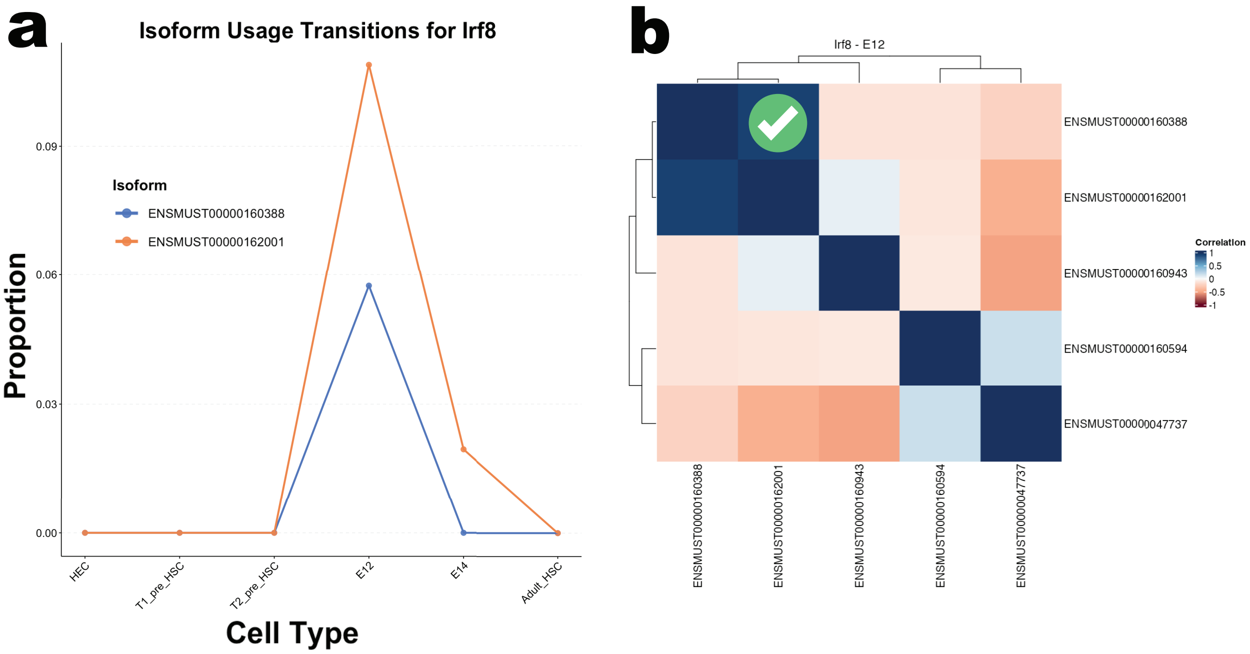

Figure SN3. Stage-specific co-expression of protein-coding and non-coding *Irf8* isoforms. (a) Isoform usage transitions across haematopoietic development stages showing synchronised peak expression of ENSMUST00000160388 (retained intron variant) and ENSMUST00000162001 (protein-coding) at the E12 stage. (b) Co-expression heatmap for E12 cells demonstrating strong positive correlation between the protein-coding and intron-retained isoforms, supporting a post-transcriptional buffering mechanism during this critical developmental stage.

SN3a). A corresponding heatmap of E12 cells provides direct evidence of the strong positive correlation in this specific context (Fig. SN3b). A structural analysis revealed a stark functional divergence: the canonical isoform (`...162001`) is a protein-coding transcript, while the co-expressed partner (`...160388`) is a non-coding transcript due to a retained intron event. The stage-specific co-expression of a functional mRNA with a non-coding variant suggests a sophisticated post-transcriptional regulatory mechanism. The biological context is critical, as embryonic day 12 in the mouse is a period of intense haematopoietic activity where the precise dosage of master regulators like *Irf8* is paramount. The literature on intron retention provides several plausible mechanisms for this buffering hypothesis:

- **Modulation of mRNA Stability via NMD:** The retained intron in `...160388` likely introduces a premature termination codon, making it a substrate for nonsense-mediated decay (NMD) [22]. By modulating splicing efficiency, the cell can control the ratio of stable, protein-coding transcripts to unstable, non-coding ones, acting as

a post-transcriptional rheostat to buffer the output of functional *Irf8* protein [23,24].

- **Nuclear Detention and Rapid Response:** The ...160388 variant may be detained in the nucleus [25,26], creating a pool of *Irf8* pre-mRNA that, upon receiving a specific developmental cue, could be rapidly spliced and exported for translation, enabling a much faster response than de novo transcription would allow.

- **Competitive Decoy for Regulatory Factors:** The non-coding ...160388 transcript could act as a competitive "sponge" or decoy for microRNAs or RNA-binding proteins that would otherwise target the functional ...162001 mRNA for repression or degradation [24,27].

Case 2: The Lineage-Variable Protein-Coding Pair

The "Mixed" pattern of the ...047737-...160943 pair was deconstructed using cell-type-specific heatmaps. The analysis revealed a positive correlation in T1_pre_HSC cells, which then inverts to a negative correlation in the subsequent T2_pre_HSC stage (Fig. SN4).

This dynamic corresponds to a switch between two distinct protein-coding isoforms: a canonical, full-length transcript (...047737) and a shorter, alternative variant (...160943) that produces a truncated protein (Fig. SN5a). The isoform switching event reflects dynamic fluctuations in relative isoform abundances rather than complete dominance shifts. Both isoforms maintain detectable expression throughout the developmental trajectory, suggesting a nuanced regulatory mechanism that continuously adjusts the balance between isoforms.

This dynamic pattern is a hallmark of functional isoform switching, a mechanism used to fine-tune a protein's activity. The literature provides a compelling mechanistic explanation for this phenomenon:

- **Precedent for Functional Isoform Switching:** Isoform switching of key transcription factors is a well-established paradigm for controlling cell fate during haematopoietic development [28]. Clinical evidence from Acute Myeloid Leukaemia (AML) demonstrates that *Irf8* isoform expression levels have prognostic significance, with both wild-type and alternatively spliced variants showing independent associations with patient outcomes [29]. Additionally, the *Irf8* transcriptional axis has been identified as a critical dependency in AML [30].

- **Dominant-Negative Activity of Truncated *Irf8*:** The most plausible mechanism is functional antagonism [31]. The truncated protein produced by ...160943 is a prime

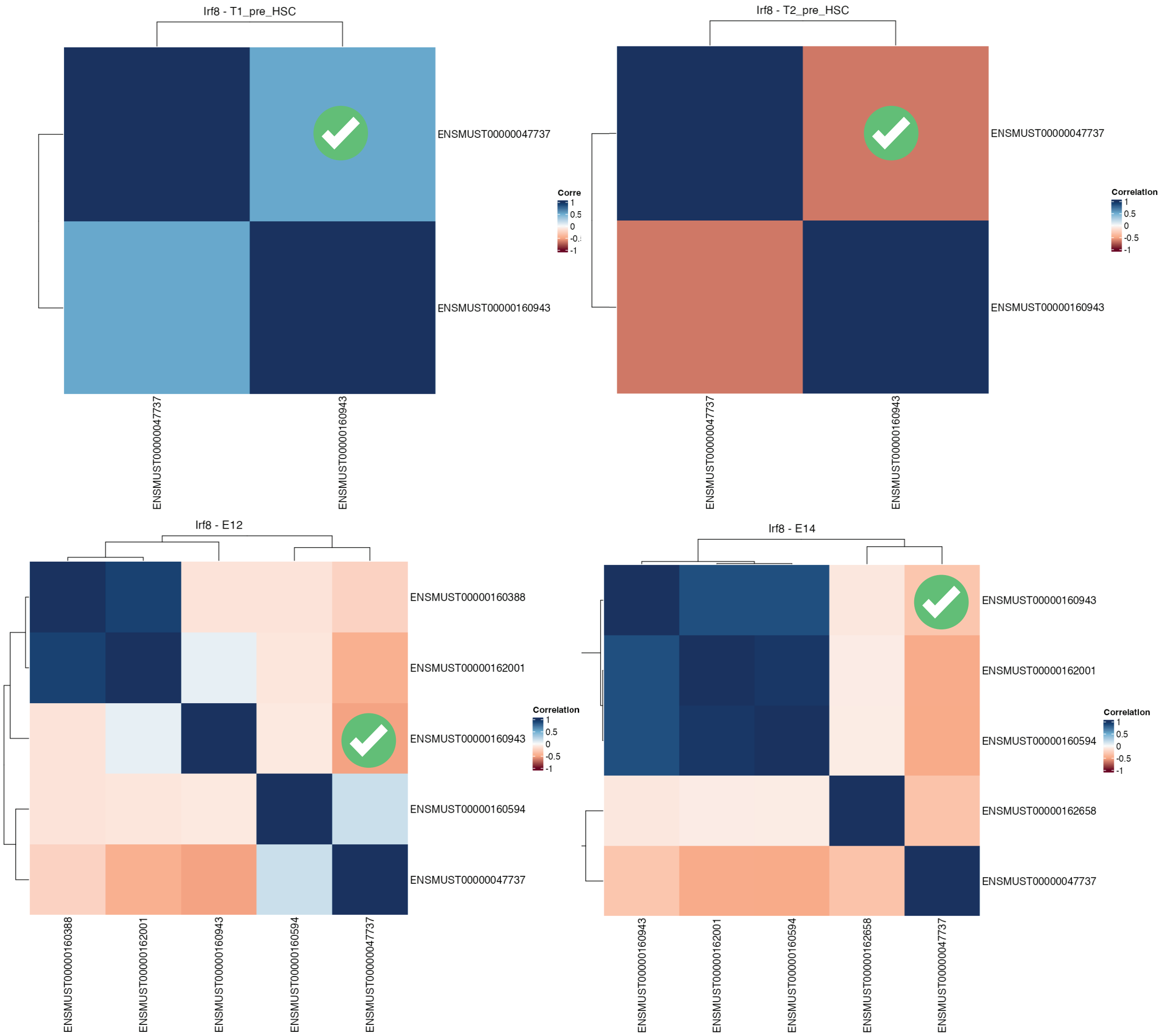

Figure SN4. Cell-type-specific co-expression heatmaps revealing dynamic correlation patterns of *Irf8* isoforms. The correlation between ENSMUST00000047737 (full-length) and ENSMUST00000160943 (truncated) shifts from positive in T1_pre_HSC stage to negative in T2_pre_HSC, E12, and E14 stages. This mixed pattern is characteristic of functional isoform switching, where the balance between activating and inhibitory isoforms is dynamically regulated during haematopoietic differentiation.

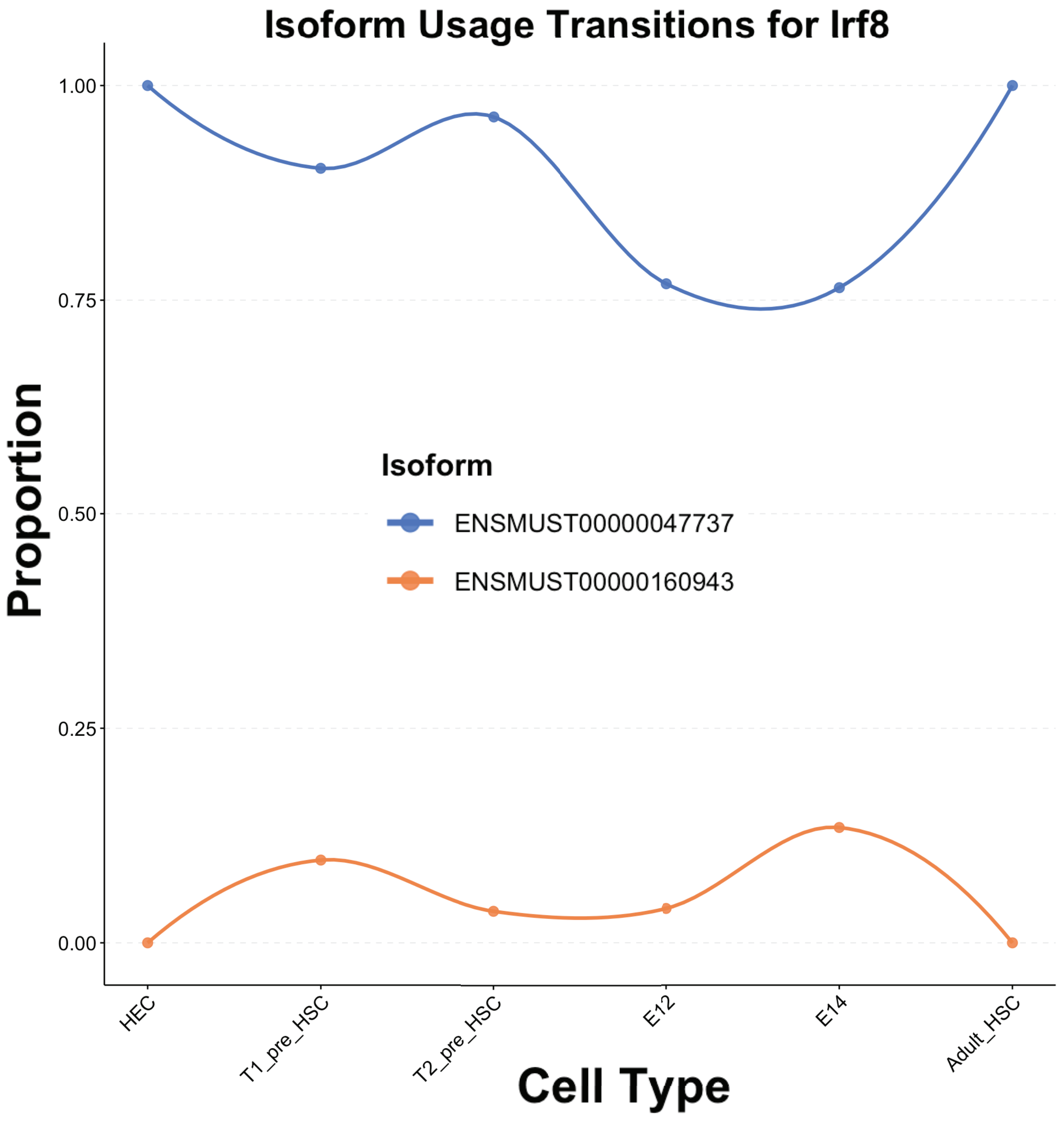

Figure SN5. Analysis of *Irf8* isoform switching dynamics. Proportional expression trajectories of ENSMUST00000047737 (canonical full-length) and ENSMUST00000160943 (truncated variant) across developmental stages, showing reciprocal expression patterns indicative of functional antagonism.

candidate to act as a dominant-negative inhibitor. Based on experimental evidence [32], if this isoform retains the protein-interaction domain but lacks a functional DNA-binding domain, it could sequester essential co-activators, thereby preventing the full-length protein from functioning effectively.

- **A "Functional Rheostat" Model:** This activator/inhibitor model explains the mixed correlation patterns. In lineages requiring high *Irf8* activity, the canonical isoform would be high while the inhibitory isoform would be low (negative correlation). Conversely, where *Irf8* activity must be dampened, the truncated isoform would be favoured. This system creates a "functional rheostat," allowing the cell to precisely titrate the net *Irf8* activity by modulating the ratio of an activator to a competitive inhibitor [31].

Conclusion and Outlook

Summary of Evidence

The analysis of the existing scientific literature provides robust support for the hypotheses derived from the observed co-expression patterns of *Irf8* isoforms. The data represent clear examples of sophisticated, multi-layered gene regulatory strategies that are fundamental to developmental biology.

- **Hypothesis 1 (Post-Transcriptional Buffering):** The stage-specific co-expression of the protein-coding isoform with the non-coding, intron-retained isoform is strongly indicative of a post-transcriptional regulatory mechanism, supported by the well-documented roles of intron retention in modulating mRNA stability [22], controlling translation through nuclear detention [25], and acting as a competitive decoy [23].

- **Hypothesis 2 (Functional Isoform Switching):** The lineage-dependent expression pattern of the canonical and truncated *Irf8* variants is consistent with the established paradigm of functional isoform switching controlling cell fate in hematopoiesis [28]. While the precise function of the truncated *Irf8* isoform is yet to be determined, its structure suggests a potential regulatory role. We hypothesise that, analogous to how alternatively spliced isoforms of other transcription factors can act as dominant-negative inhibitors, the truncated *Irf8* variant may modulate the activity of the full-length protein. This isoform interplay could represent a "rheostat" mechanism for fine-tuning *Irf8*'s potent regulatory activity, which is known to be critical in myeloid leukemia [29,30].

Implications for Understanding Haematopoietic Development

These findings underscore the critical importance of performing analyses at the isoform level. A simple gene-level quantification of *Irf8* would have completely obscured these intricate regulatory dynamics. The biological and clinical significance lies not just in the total amount of *Irf8* transcript but in the specific ratio and interplay of its various isoforms.

References

[22] Wong, J.J.-L., Ritchie, W., Ebner, O.A., Selbach, M., Wong, J.W.H., Huang, Y., Gao, D., Pinello, N., Gonzalez, M., Baidya, K., Thoeng, A., Khoo, T.-L., Bailey, C.G., Holst, J., Rasko, J.E.J.: Orchestrated intron retention regulates normal granulocyte differentiation. Cell **154**, 583–595 (2013)

[23] Braunschweig, U., Barbosa-Morais, N.L., Pan, Q., Nachman, E.N., Alipanahi, B., Gonatopoulos-Pournatzis, T., Frey, B., Irimia, M., Blencowe, B.J.: Widespread intron retention in mammals functionally tunes transcriptomes. Genome Research **24**, 1774–1786 (2014)

[24] Monteuuis, G., Wong, J.J.L., Bailey, C.G., Schmitz, U., Rasko, J.E.J.: The changing paradigm of intron retention: regulation, ramifications and recipes. Nucleic Acids Research **47**, 11497–11513 (2019)

[25] Schmitz, U., Pinello, N., Jia, F., Alasmari, S., Ritchie, W., Keightley, M.-C., Shini, S., Lieschke, G.J., Wong, J.J.-L., Rasko, J.E.J.: Intron retention enhances gene regulatory complexity in vertebrates. Genome Biology **18**, 216 (2017)

[26] Rekosh, D., Hammarskjold, M.-L.: Intron retention in viruses and cellular genes: Detention, border controls and passports. WIREs RNA **9**(3), 1470 (2018)

[27] Thomson, D.W., Dinger, M.E.: Endogenous microRNA sponges: evidence and controversy. Nature Reviews Genetics **17**, 272–283 (2016)

[28] Chen, S., Abdel-Wahab, O.: Splicing regulation in hematopoiesis. Current Opinion in Hematology **28**(4) (2021)

[29] Pogosova-Agadjanyan, E.L., Kopecky, K.J., Ostronoff, F., Appelbaum, F.R., Godwin, J., Lee, H., List, A.F., May, J.J., Oehler, V.G., Petersdorf, S., Pogosov, G.L., Radich, J.P., Willman, C.L., Meshinchi, S., Stirewalt, D.L.: The prognostic significance of IRF8

transcripts in adult patients with acute myeloid leukemia. PLOS ONE **8**(8), 70812 (2013)

[30] Cao, Z., Budinich, K.A., Huang, H., Ren, D., Lu, B., Zhang, Z., Chen, Q., Zhou, Y., Huang, Y.-H., Alikarami, F., Kingsley, M.C., Lenard, A.K., Wakabayashi, A., Khandros, E., Bailis, W., Qi, J., Carroll, M.P., Blobel, G.A., Faryabi, R.B., Bernt, K.M., Berger, S.L., Shi, J.: ZMYND8-regulated IRF8 transcription axis is an acute myeloid leukaemia dependency. Molecular Cell **81**, 3604–3622 (2021)

[31] Lambourne, L., Mattioli, K., Santoso, C., Sheynkman, G., Inukai, S., Kaundal, B., Berenson, A., Spirohn-Fitzgerald, K., Bhattacharjee, A., Rothman, E., Shrestha, S., Laval, F., Carroll, B.S., Plassmeyer, S.P., Emenecker, R.J., Yang, Z., Bisht, D., Sewell, J.A., Li, G., Prasad, A., Phanor, S., Lane, R., Moyer, D.C., Hunt, T., Balcha, D., Gebbia, M., Twizere, J.-C., Hao, T., Holehouse, A.S., Frankish, A., Riback, J.A., Salomonis, N., Calderwood, M.A., Hill, D.E., Sahni, N., Vidal, M., Bulyk, M.L., Fuxman Bass, J.I.: Widespread variation in molecular interactions and regulatory properties among transcription factor isoforms. Molecular Cell **85**, 1341–1359 (2025)

[32] Belaguli, N.S., Zhou, W., Trinh, T.-H.T., Majesky, M.W., Schwartz, R.J.: Dominant negative murine serum response factor: alternative splicing within the activation domain inhibits transactivation of serum response factor binding targets. Molecular and Cellular Biology **19**, 4582–4591 (1999)
