# Supplementary material for: ScIsoX: a multidimensional framework for measuring isoform-level transcriptomic complexity in single cells
Source: Genome Biol. 2025 Sep 22;26:289. doi: 10.1186/s13059-025-03758-5 (PMC12455757; doi:10.1186/s13059-025-03758-5)
Supplement: Supplementary file 4 — Additional file 4. QC_Report_Blood_Data.html. [file 13059_2025_3758_MOESM4_ESM.html]

ScIsoX Quality Control Report


# ScIsoX Quality Control Report

Generated on 2025-07-27 | ScIsoX v1.1.0

Genes in SCHT

1,936

Cells after QC

181

Total Isoforms

7,327

SCHT Sparsity

67.9%

## Input Data Characteristics

**Input Data Type:** Raw Count Matrices

| Metric | Value |
| --- | --- |
| Original genes | 55,487 |
| Original transcripts | 142,238 |
| Original cells | 205 |
| Gene matrix sparsity | 86.80% |
| Transcript matrix sparsity | 91.57% |
| Median genes/cell | 7,368 |
| Median transcripts/cell | 11,943 |

### Cell Type Distribution

- Number of cell types: 7

| Cell Type | Count | Percentage |
| --- | --- | --- |
| Adult\_HSC | 44 | 21.7% |
| AEC | 18 | 8.9% |
| E12 | 21 | 10.3% |
| E14 | 32 | 15.8% |
| HEC | 24 | 11.8% |
| T1\_pre\_HSC | 26 | 12.8% |
| T2\_pre\_HSC | 38 | 18.7% |

## QC Parameters

| Parameter | Applied Value | MAD Strategy | Interval 90 | Interval 80 |
| --- | --- | --- | --- | --- |
| Min genes per cell | 4000 | 980 | 4106 | 4474 |
| Max genes per cell | 10000 | 13756 | 10648 | 9663 |
| Min cells expressing | 2.0% | - | - | - |
| Min expression | 1.0e-06 | - | - | - |

### Strategy Explanations:

- MAD Strategy: Uses median ± 3 MAD, reduces risk of including poor quality cells whilst maintaining robustness to outliers
- Interval 90: Uses 5th and 95th percentiles, balances stringency with dataset preservation
- Interval 80: Uses 10th and 90th percentiles, provides more aggressive filtering for higher quality cell selection

## Filtering Summary

| Category | Count Removed |
| --- | --- |
| Genes | 37,606 |
| Transcripts | 0 |
| Cells | 24 |

### Cell Removal Reasons

| Reason | Cell Count | Percentage |
| --- | --- | --- |
| Too few genes | 8 | 3.9% |
| Too many genes | 17 | 8.3% |

## Highly Variable Gene Selection

| Metric | Value |
| --- | --- |
| HVGs requested | 3,000 |
| HVGs selected | 3,000 |

### HVG Filtering Details

| Description | Count | Status |
| --- | --- | --- |
| Total genes available after QC | 17,881 | - |
| HVGs with single isoform | 1,021 | Removed |
| HVGs with multiple isoforms | 1,936 | Kept |
| Percentage multi-isoform HVGs | 64.5% | - |
| Final genes in SCHT | 1,936 | - |

## SCHT Structure

| Metric | Value |
| --- | --- |
| Genes in SCHT | 1,936 |
| Cells after QC | 181 |
| Total isoforms | 7,327 |
| Max isoforms per gene | 21 |
| Mean isoforms per gene | 3.78 |

## Sparsity Analysis

### Comprehensive Sparsity Comparison

| Matrix Type | Elements | Non-zeros | Zeros | Sparsity % |
| --- | --- | --- | --- | --- |
| Original Transcript Matrix | 29,158,790 | 2,457,789 | 26,701,001 | 91.57% |
| Filtered Matrix (Post-QC) | 1,326,187 | 73,057 | 1,253,130 | 94.49% |
| Naive 3D Tensor | 7,358,736 | 73,057 | 7,285,679 | 99.01% |
| SCHT Structure | 227,837 | 73,057 | 154,780 | 67.93% |

### Zero Padding Reduction

| Comparison | Zero Elements Avoided |
| --- | --- |
| vs Original Matrix | 26,546,221 |
| vs Filtered Matrix | 1,098,350 |
| vs Naive 3D Tensor | 7,130,899 |

## Performance Metrics

| Metric | Value |
| --- | --- |
| Total processing time | 47.10 seconds (0.79 minutes) |
| Memory used | 181.10 MB |

Generated on 2025-07-27 using ScIsoX v1.1.0

Single-cell Transcriptomic Complexity Analysis
