# Supplementary material for: ScIsoX: a multidimensional framework for measuring isoform-level transcriptomic complexity in single cells
Source: Genome Biol. 2025 Sep 22;26:289. doi: 10.1186/s13059-025-03758-5 (PMC12455757; doi:10.1186/s13059-025-03758-5)
Supplement: Supplementary file 5 — Additional file 5. QC_Report_Brain_Data.html. [file 13059_2025_3758_MOESM5_ESM.html]

ScIsoX Quality Control Report


# ScIsoX Quality Control Report

Generated on 2025-07-27 | ScIsoX v1.1.0

Genes in SCHT

2,248

Cells after QC

272

Total Isoforms

8,637

SCHT Sparsity

65.9%

## Input Data Characteristics

**Input Data Type:** Raw Count Matrices

| Metric | Value |
| --- | --- |
| Original genes | 31,335 |
| Original transcripts | 132,119 |
| Original cells | 301 |
| Gene matrix sparsity | 70.29% |
| Transcript matrix sparsity | 85.92% |
| Median genes/cell | 9,768 |
| Median transcripts/cell | 17,791 |

### Cell Type Distribution

- Number of cell types: 7

| Cell Type | Count | Percentage |
| --- | --- | --- |
| Astro | 43 | 10.9% |
| ExciteNeuron | 66 | 16.7% |
| Immune | 44 | 11.1% |
| InhibNeuron | 39 | 9.9% |
| Oligo | 105 | 26.6% |
| Progenitor | 30 | 7.6% |
| Vasc | 68 | 17.2% |

## QC Parameters

| Parameter | Applied Value | MAD Strategy | Interval 90 | Interval 80 |
| --- | --- | --- | --- | --- |
| Min genes per cell | 2200 | 100 | 2297 | 3685 |
| Max genes per cell | 15000 | 21858 | 14861 | 13851 |
| Min cells expressing | 2.0% | - | - | - |
| Min expression | 1.0e-06 | - | - | - |

### Strategy Explanations:

- MAD Strategy: Uses median ± 3 MAD, reduces risk of including poor quality cells whilst maintaining robustness to outliers
- Interval 90: Uses 5th and 95th percentiles, balances stringency with dataset preservation
- Interval 80: Uses 10th and 90th percentiles, provides more aggressive filtering for higher quality cell selection

## Filtering Summary

| Category | Count Removed |
| --- | --- |
| Genes | 11,269 |
| Transcripts | 0 |
| Cells | 29 |

### Cell Removal Reasons

| Reason | Cell Count | Percentage |
| --- | --- | --- |
| Too few genes | 15 | 5.0% |
| Too many genes | 15 | 5.0% |

## Highly Variable Gene Selection

| Metric | Value |
| --- | --- |
| HVGs requested | 3,000 |
| HVGs selected | 3,000 |

### HVG Filtering Details

| Description | Count | Status |
| --- | --- | --- |
| Total genes available after QC | 20,066 | - |
| HVGs with single isoform | 752 | Removed |
| HVGs with multiple isoforms | 2,248 | Kept |
| Percentage multi-isoform HVGs | 74.9% | - |
| Final genes in SCHT | 2,248 | - |

## SCHT Structure

| Metric | Value |
| --- | --- |
| Genes in SCHT | 2,248 |
| Cells after QC | 272 |
| Total isoforms | 8,637 |
| Max isoforms per gene | 16 |
| Mean isoforms per gene | 3.84 |

## Sparsity Analysis

### Comprehensive Sparsity Comparison

| Matrix Type | Elements | Non-zeros | Zeros | Sparsity % |
| --- | --- | --- | --- | --- |
| Original Transcript Matrix | 39,767,819 | 5,598,505 | 34,169,314 | 85.92% |
| Filtered Matrix (Post-QC) | 2,349,264 | 152,738 | 2,196,526 | 93.50% |
| Naive 3D Tensor | 9,783,296 | 152,738 | 9,630,558 | 98.44% |
| SCHT Structure | 447,825 | 152,738 | 295,087 | 65.89% |

### Zero Padding Reduction

| Comparison | Zero Elements Avoided |
| --- | --- |
| vs Original Matrix | 33,874,227 |
| vs Filtered Matrix | 1,901,439 |
| vs Naive 3D Tensor | 9,335,471 |

## Performance Metrics

| Metric | Value |
| --- | --- |
| Total processing time | 49.84 seconds (0.83 minutes) |
| Memory used | 351.50 MB |

Generated on 2025-07-27 using ScIsoX v1.1.0

Single-cell Transcriptomic Complexity Analysis
