# Supplementary material for: ScIsoX: a multidimensional framework for measuring isoform-level transcriptomic complexity in single cells
Source: Genome Biol. 2025 Sep 22;26:289. doi: 10.1186/s13059-025-03758-5 (PMC12455757; doi:10.1186/s13059-025-03758-5)
Supplement: Supplementary file 6 — Additional file 6. QC_Report_PBMC_Data.html. [file 13059_2025_3758_MOESM6_ESM.html]

ScIsoX Quality Control Report


# ScIsoX Quality Control Report

Generated on 2025-07-27 | ScIsoX v1.1.0

Genes in SCHT

2,980

Cells after QC

11,640

Total Isoforms

167,204

SCHT Sparsity

98.3%

## Input Data Characteristics

**Input Data Type:** Raw Count Matrices

| Metric | Value |
| --- | --- |
| Original genes | 27,176 |
| Original transcripts | 792,617 |
| Original cells | 12,852 |
| Gene matrix sparsity | 96.80% |
| Transcript matrix sparsity | 99.87% |
| Median genes/cell | 814 |
| Median transcripts/cell | 922.5 |

### Cell Type Distribution

- Number of cell types: 26

| Cell Type | Count | Percentage |
| --- | --- | --- |
| Astrocyte | 2 | 0.0% |
| B\_cell | 776 | 6.1% |
| BM | 370 | 2.9% |
| BM & Prog. | 8 | 0.1% |
| CMP | 38 | 0.3% |
| DC | 5 | 0.0% |
| Epithelial\_cells | 4 | 0.0% |
| Erythroblast | 4 | 0.0% |
| Gametocytes | 1 | 0.0% |
| GMP | 62 | 0.5% |
| HSC\_-G-CSF | 1,626 | 12.8% |
| HSC\_CD34+ | 18 | 0.1% |
| Keratinocytes | 2 | 0.0% |
| Macrophage | 74 | 0.6% |
| MEP | 6 | 0.0% |
| Monocyte | 1,162 | 9.1% |
| Myelocyte | 116 | 0.9% |
| Neurons | 1 | 0.0% |
| Neutrophils | 190 | 1.5% |
| NK\_cell | 992 | 7.8% |
| Platelets | 236 | 1.9% |
| Pre-B\_cell\_CD34- | 749 | 5.9% |
| Pro-B\_cell\_CD34+ | 26 | 0.2% |
| Pro-Myelocyte | 11 | 0.1% |
| T\_cells | 6,226 | 49.0% |

## QC Parameters

| Parameter | Applied Value | MAD Strategy | Interval 90 | Interval 80 |
| --- | --- | --- | --- | --- |
| Min genes per cell | 240 | 100 | 243 | 293 |
| Max genes per cell | 1900 | 1820 | 1850 | 1566 |
| Min cells expressing | 2.0% | - | - | - |
| Min expression | 1.0e-06 | - | - | - |

### Strategy Explanations:

- MAD Strategy: Uses median ± 3 MAD, reduces risk of including poor quality cells whilst maintaining robustness to outliers
- Interval 90: Uses 5th and 95th percentiles, balances stringency with dataset preservation
- Interval 80: Uses 10th and 90th percentiles, provides more aggressive filtering for higher quality cell selection

## Filtering Summary

| Category | Count Removed |
| --- | --- |
| Genes | 19,227 |
| Transcripts | 0 |
| Cells | 1,212 |

### Cell Removal Reasons

| Reason | Cell Count | Percentage |
| --- | --- | --- |
| Too few genes | 603 | 4.7% |
| Too many genes | 567 | 4.4% |

## Highly Variable Gene Selection

| Metric | Value |
| --- | --- |
| HVGs requested | 3,000 |
| HVGs selected | 3,000 |

### HVG Filtering Details

| Description | Count | Status |
| --- | --- | --- |
| Total genes available after QC | 7,949 | - |
| HVGs with single isoform | 20 | Removed |
| HVGs with multiple isoforms | 2,980 | Kept |
| Percentage multi-isoform HVGs | 99.3% | - |
| Final genes in SCHT | 2,980 | - |

## SCHT Structure

| Metric | Value |
| --- | --- |
| Genes in SCHT | 2,980 |
| Cells after QC | 11,640 |
| Total isoforms | 167,204 |
| Max isoforms per gene | 268 |
| Mean isoforms per gene | 56.11 |

## Sparsity Analysis

### Comprehensive Sparsity Comparison

| Matrix Type | Elements | Non-zeros | Zeros | Sparsity % |
| --- | --- | --- | --- | --- |
| Original Transcript Matrix | 10,186,713,684 | 13,127,059 | 10,173,586,625 | 99.87% |
| Filtered Matrix (Post-QC) | 1,946,254,560 | 1,356,892 | 1,944,897,668 | 99.93% |
| Naive 3D Tensor | 9,296,169,600 | 1,356,892 | 9,294,812,708 | 99.99% |
| SCHT Structure | 79,856,344 | 1,356,892 | 78,499,452 | 98.30% |

### Zero Padding Reduction

| Comparison | Zero Elements Avoided |
| --- | --- |
| vs Original Matrix | 10,095,087,173 |
| vs Filtered Matrix | 1,866,398,216 |
| vs Naive 3D Tensor | 9,216,313,256 |

## Performance Metrics

| Metric | Value |
| --- | --- |
| Total processing time | 764.96 seconds (12.75 minutes) |
| Memory used | 2118.30 MB |

Generated on 2025-07-27 using ScIsoX v1.1.0

Single-cell Transcriptomic Complexity Analysis
